# Charge‐Assisted Halogen Bonding in an Ionic Cavity of a Coordination Cage Based on a Copper(I) Iodide Cluster

**DOI:** 10.1002/anie.202215689

**Published:** 2023-01-11

**Authors:** Anssi Peuronen, Anni I. Taponen, Elina Kalenius, Ari Lehtonen, Manu Lahtinen

**Affiliations:** ^1^ Department of Chemistry University of Turku 20014 Turku Finland; ^2^ Department of Chemistry University of Jyvaskyla P.O. Box 35 40014 Jyvaskyla Finland

**Keywords:** Cluster Compounds, Coordination Cage, Halogen Bond, Host-Guest Systems, Supramolecular Chemistry

## Abstract

The design of molecular containers capable of selectively binding specific guest molecules presents an interesting synthetic challenge in supramolecular chemistry. Here, we report the synthesis and structure of a coordination cage assembled from Cu_3_I_4_^−^ clusters and tripodal cationic N‐donor ligands. Owing to the localized permanent charges in the ligand core the cage binds iodide anions in specific regions within the cage through ionic interactions. This allows the selective binding of bromomethanes as secondary guest species within the cage promoted by halogen bonding, which was confirmed by single‐crystal X‐ray diffraction.

## Graphical abstract

An unusual Cu_3_I_4_^−^ based coordination cage was synthesized by a reaction between copper (I) iodide and a tripodal cationic ligand. X‐ray structural evidence demonstrates that this cage can selectively encapsulate bromomethanes by concerted ligand‐iodide‐bromomethane ionic and halogen bonding interactions.

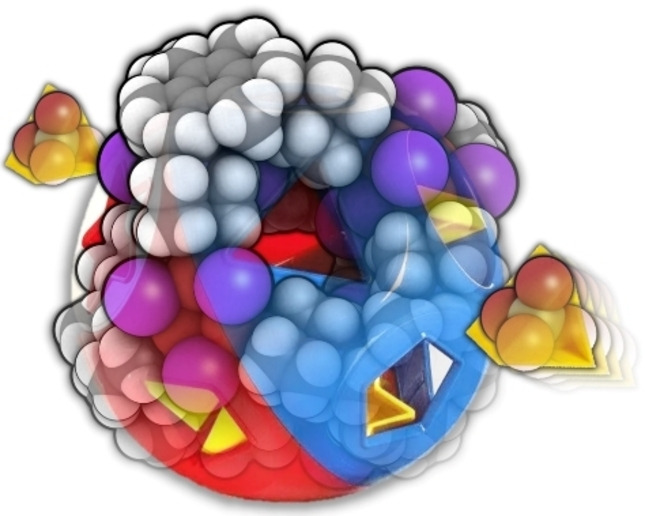

## Introduction

Multi‐component methods of construction are powerful synthetic approaches that in the case of coordination‐driven systems rely on complementary geometrical features and binding modes of the ligand and metal counterparts. This has manifested in rapid expansion of new topologies of supramolecular hosts which possess well‐defined structures and internal cavities that enable the inclusion of other molecules and ions. Such systems have implications in numerous important fields such as reactivity and catalysis, molecular capture, and biomedicine.[^1^, ^2^] Importantly, it has also facilitated the tailoring of synthetic cavities as demonstrated, for example, by the rich endohedral chemistry of modified tetrahedral M_4_L_6_[^3^, ^4^, ^5^, ^6^, ^7^] and cuboctahedral M_12_L_24_ architectures.^[8]^

While the use of single metal ions as connecting nodes between the ligands is a typical design feature of coordination cages, the construction of cages using various metal clusters is also an established strategy. This approach can be advantageous in several ways including the possibility to synthesize new cage structures, facilitated by the often more diverse metal‐ligand connectivity of clusters compared to single metal ions, an increase in size leading to larger inner pore diameters and cage openings and inheritance of properties related to the clusters. Cluster‐derived cages have displayed potential in fields of optical sensing, catalysis, and magnetism, for example.^[9]^ Copper(I) iodide clusters are known for their rich structural chemistry and interesting photophysical properties.[^10^, ^11^, ^12^, ^13^, ^14^] Copper iodide is particularly well known for its tendency to form Cu_4_I_4_ cubane‐like clusters in the presence of N‐, P‐, or S‐donor ligands.[^11^, ^15^, ^16^] However, changes in ligand structure, denticity, stoichiometry, reaction solvent, or temperature readily invoke isomerism or give other Cu^I^_*n*_I_*m*_^*n*−*m*^ clusters with various structures and composition.[^13^, ^14^, ^16^, ^17^] As a consequence of their structural adaptability and spectroscopic properties, clusters based on copper(I) halides have been employed as building blocks in metal‐organic materials with molecular as well as extended structures.[^18^, ^19^, ^20^, ^21^] Nevertheless, to our knowledge, discrete Cu^I^_*n*_I_*m*_^*n*−*m*^ cluster based coordination cages remain unexplored.

**Scheme 1:**
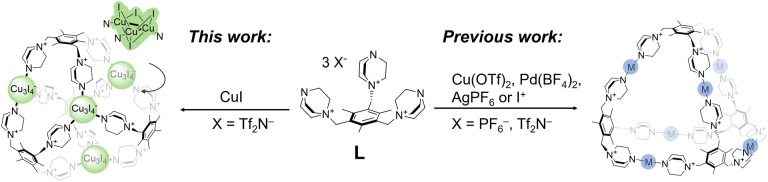
An overview of isostructural coordination cages derived from ligand **L** ^3+^ and metal or I^+^ cations (right) compared to the [Cu_3_I_4_]^−^ cluster‐based cage presented in this work (left).

One of our ongoing interests is the study of supramolecular assemblies using cationic ligands as the molecular backbones. In this context, we have discovered 1,4‐diazabicyclo[2.2.2]octane (DABCO) based cationic ligand **L** ^3+^ (Scheme 1) as a useful building block of various supramolecular systems ranging from hydrogen‐bonded capsules to coordination and halonium cages[^22^, ^23^, ^24^] (Scheme 1). **L** ^3+^ is a tritopic N‐donor ligand susceptible to adopt a bowl‐shaped all‐*cis* conformation in the presence of anions that are able to protrude and bind into its tricationic cleft. Garratt and co‐workers already provided crystallographic evidence of this behavior in the late 1990s by using **L** ^3+^ as a cationic host for [Fe(CN)_6_]^3−^.^[25]^ Our more recent findings have provided further such support and demonstrated that by using **L** ^3+^ as a ligand, highly cationic molecular cages with specific anion‐binding sites could be synthesized.

Since the realization of the robust supramolecular **L** ^3+^⋅⋅⋅anion motif, our effort has been focused on utilizing the cationic ligand in the synthesis of *endo*‐functionalized cages in which this ionic interaction would be used for molecular recognition of secondary guest molecules by the combination of ionic and hydrogen (HB) or halogen bond (XB) interactions. There is extensive evidence on the benefits of *endo*‐functionalization in terms of selective and strong binding between the host and guest.[^6^, ^7^, ^26^, ^27^, ^28^] In the context of cationic receptors, Severin and co‐workers have demonstrated how three closely positioned piperazinium moieties in a coordination‐driven helicate contribute to the recognition of phosphate and acetate in water.^[29]^ Nitschke et al. have recently shown the importance of cationic paneling in a water‐soluble anion receptor cage,^[30]^ whereas metallacycles derived from ditopic 4,4′‐bipyridinium or 2,7‐diazapyrenium ligands have been shown to give rise to catenates with crown ethers.[^31^, ^32^, ^33^, ^34^, ^35^, ^36^] Up until now, our efforts to utilize **L** ^3+^ in this manner have been hindered by challenges in finding a suitable combination of metal node and anion that would together provide an appropriate cage scaffold and a suitable anionic endohedral environment with enough space to accommodate an additional guest molecule. In this work, we report a cluster‐based approach of building a coordination cage from **L** ^3+^ and a rarely observed Cu_3_I_4_^−^ cluster as well as the selective binding of bromomethanes through **L** ^3+^⋅⋅⋅I^−^⋅⋅⋅Br−R interactions.

## Results and Discussion

**Figure 1:**
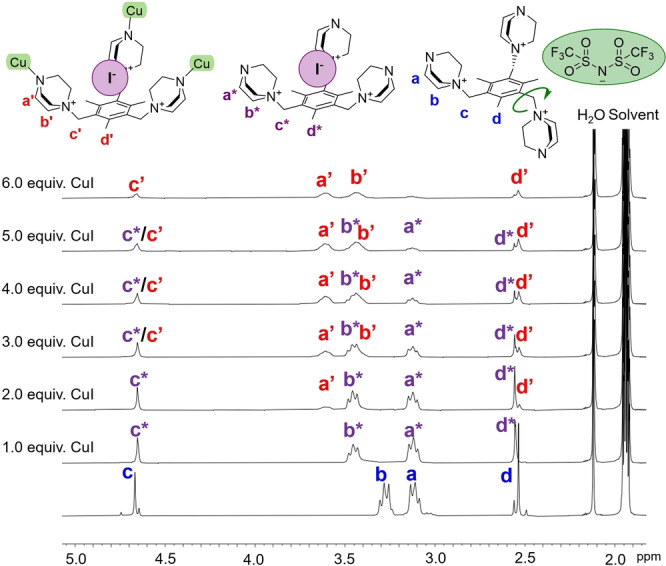
Complexation between **L**(Tf_2_N)_3_ and copper(I) iodide monitored by ^1^H NMR spectroscopy in CD_3_CN. The loss of signal intensity arises from precipitation of the cage species from solution.

The reaction between copper(I) iodide and **L**(Tf_2_N)_3_, i.e. the bis(trifluoromethylsulfonyl)imide salt of ligand **L** ^3+^, was initially monitored by ^1^H NMR spectroscopy (Figure 1). The addition of one equivalent of copper(I) iodide into a CD_3_CN solution of **L**(Tf_2_N)_3_ results in a significant shift of the methylene group protons adjacent to the quaternary ammonium centers of the DABCO moiety. Also, the mesitylene CH_2_ and CH_3_ signals, which suggest that several conformational isomers co‐exist with **L**(Tf_2_N)_3_ in solution, become well resolved. This corresponds to the strong binding of the iodide anion in the cationic pocket of **L** ^3+^ and, thus, stabilization of the all‐*cis* conformation. This was further verified by an additional experiment using tetrabutylammonium iodide instead of CuI as the source of the I^−^ anion, which provided a ^1^H NMR spectrum where the methylene shifts are identical to that from a 1 : 1 reaction with CuI (Figure S1). We were also able to confirm this by single‐crystal X‐ray determination of the structure of **L**(Tf_2_N)_2_I, which shows a single iodide resides in the cationic pocket of **L** ^3+^ (Figure S2, Table S4).^[37]^ In light of these results it is reasonable to assume that the first step of the reaction between **L**(Tf_2_N)_3_ and CuI involves the formation of an **L** ^3+^⋅⋅⋅I^−^ complex while the addition of second equivalent of CuI gives rise to a new set of signals, which upon further addition of CuI become more prominent. Ultimately, after the addition of six equivalents of CuI, the monitored signals almost completely disappear because of precipitation of the product from CD_3_CN. Diffusion‐ordered NMR spectroscopy (DOSY) measurements of this species in a 3 : 1 mixture of CD_3_CN/DMF‐d7 gave a diffusion coefficient (*D*) of 4.82×10^−10^ m^2^ s^−1^, which corresponds to a spherical diameter of ca. 2.2 nm according to the Stokes–Einstein equation (Supporting Information). This value correlates well with the different M_6_ **L**_4_ type complexes we have studied earlier,^[24]^ and thus suggests the formation of a cage species of comparable size.

**Figure 2:**
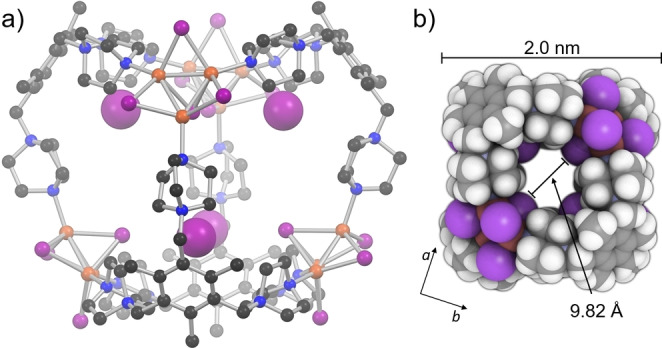
a) Illustration of X‐ray structure of [**L**_4_(Cu_3_I_4_)_4_]^8+^ cage with the encapsulated endohedral iodide anions drawn as larger spheres. b) Space‐filling model of the cage viewed along the crystallographic *c*‐axis with the diameters of the cage as well as its cavity shown.

Colorless single crystals of the putative cage compound were obtained from CH_3_CN using a 6 : 1 mixture of CuI and **L**(Tf_2_N)_3_ based on NMR analyses and, subsequently, the structure determination was successfully conducted, which showed the compound belonged to the tetragonal space group $I\bar{4}$ . The crystal structure^[37]^ reveals the formation of a discrete tetrahedral cage species consisting of four **L** ^3+^ ligands and four unusual Cu_3_I_4_^−^ clusters, namely [**L**_4_(Cu_3_I_4_)_4_]^8+^ (Figure 2). The Cu_3_I_4_^−^ cluster, which appears in only three prior examples in the Cambridge Structural Database (CSD references: ITASAX, VIDFET/VIDFIX, VUXWAP),^[38]^ bears close resemblance to the Cu_4_I_4_ cubane after removal of one of the cornering Cu^I^ ions. This is well illustrated in the comparison of [**L**_4_(Cu_3_I_4_)_4_]^8+^ and crystallographic data of 61 Cu_4_I_4_ N‐donor complexes obtained from the CSD (Figure S4). The structural similarity is also reflected in the photoluminescent properties of the Cu_3_I_4_^−^ cluster, as the shape of the excitation/emission spectra of the isolated compound closely resembles that of the Cu_4_I_4_ cluster. The excitation peak maximum is blue‐shifted ca. 40 nm, whereas the emission peak maximum lies at 540 nm, i.e. at a wavelength ca. 50 nm lower than those of Cu_4_I_4_ clusters connected through DABCO ligands (Figure S5).^[39]^ In [**L**_4_(Cu_3_I_4_)_4_]^8+^ the Cu_3_I_4_^−^ clusters each coordinate to three distinct ligands and thus both the clusters as well as the **L** ^3+^ ligands act as trigonal nodes of the cage. The resulting cage, therefore, has six trigonal vertices and six openings (ca. 6.8×7.4 Å across), which gives a geometry that can be described as a distorted cube. The maximum external diameter of the cage, 2.1 nm, calculated from the single‐crystal X‐ray data, corresponds well to the spherical diameter of the cage obtained by DOSY. As predicted, the ligands adopt an all‐ *cis*‐type conformation, and each encloses a single iodide anion in the space between the three cationic DABCO groups, thereby resulting in the encapsulation of a total of four I^−^ anions as guests within the cage, namely 4 I^−^@[**L**_4_(Cu_3_I_4_)_4_]^8+^. The remainder of the crystal lattice comprises exohedral iodides as well as endo‐ and exohedral disordered iodocuprates of unresolved exact composition (due to symmetry‐related disorder) together with disordered solvent molecules.

It is noteworthy that the self‐assembly reaction proceeds in a similar manner using a stoichiometric amount of CuI and a secondary iodide source. Indeed, according to ^1^H NMR spectroscopy, the reaction of 1 : 3 : 3 equivalents of **L**(Tf_2_N)_3_, CuI, and tetrabutylammonium iodide also gives the desired cage quantitatively, which was also verified by SCXRD. The validity of the two different synthetic routes were also confirmed by ESI‐MS. The in situ prepared solution of **L**(Tf_2_N)_3_, CuI, and tetramethylammonium iodide in a 1 : 3 : 3 ratio in MeCN as well as an isolated powder precipitated from a solution of CuI and **L**(Tf_2_N)_3_ in a 6 : 1 ratio by the addition of diethyl ether were both measured in the positive ion mode. Both samples show the expected [**L**_4_(Cu_3_I_4_)_4_(I)_4_]^4+^ species (*m*/*z*_exp_ 1320.80) together with a set of different triply charged species, which were identified as [**L**_4_(Cu_3_I_4_)_4_(I)_5_]^3+^ (*m*/*z*_exp_ 1803.53), [**L**_4_(Cu_3_I_4_)_4_(I)_4_(Tf_2_N)]^3+^ (*m*/*z*_exp_ 1854.52), and [**L**_4_(Cu_3_I_4_)_4_(I)_5_(CuI)]^3+^ (*m*/*z*_exp_ 1867.19, see further details in Supporting Information, Tables S2 and S3 and Figure S6). Notably, the ESI‐MS also shows the presence of various degradation products of the [**L**_4_(Cu_3_I_4_)_4_]^8+^ cage, thus suggesting that the cage is not particularly stable under the applied ionization conditions.

**Figure 3:**
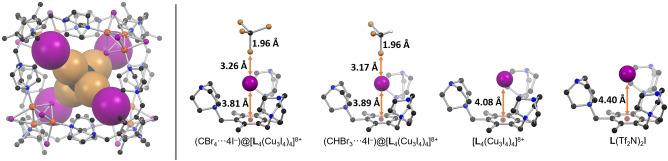
Left: (CBr_4_⋅⋅⋅4I^−^)@[**L**_4_(Cu_3_I_4_)_4_]^8+^ host–guest complex with the guest species highlighted by a space‐filling model. Right: comparison of the crystal structure derived ionic and halogen bond parameters of the different host–guest systems investigated herein. Only the **L** ^3+^⋅⋅⋅guest(s) unit of each structure is shown. For the two bromomethane host–guest complexes, the reported distances are calculated as averages of the two distinct molecular species that exist in the respective crystal structures.

The size of the interstitial space created within the 4I^−^@[**L**_4_(Cu_3_I_4_)_4_]^8+^ host–guest complex between the iodide guests and the Cu_3_I_4_^−^ clusters is large enough to accommodate small secondary guest molecules of suitable shape and size (cage centroid⋅⋅⋅I^−^_guest_ distance is 4.9 Å). Furthermore, we realized that halogen bonds (XB)—i.e. interactions in which the halogen atom X acts as an electrophilic species towards an electron donor atom Y in an R−X⋅⋅⋅Y fashion^[40]^—could be used as the driving force for secondary guest encapsulation within 4I^−^@[**L**_4_(Cu_3_I_4_)_4_]^8+^. Crystallization of the [**L**_4_(Cu_3_I_4_)_4_]^8+^ cage in the presence of carbon tetrabromide (CBr_4_) results in the formation of prismatic crystals, which were solved in the tetragonal space group $P\bar{4}$ with two quarters of the distinct cages in the asymmetric unit. According to the structural parameters, the [**L**_4_(Cu_3_I_4_)_4_]^8+^ cage itself remains relatively unchanged compared to the parent cage, but the space within the endohedral iodides is occupied by a single CBr_4_ molecule which resides well within Br⋅⋅⋅I vdW range (3.83 Å) for all four iodide anions through C−Br⋅⋅⋅I^−^ halogen bonds (Figure 3). The Br⋅⋅⋅I^−^ distances range from 3.26 Å to 3.27 Å, which are very similar to the XB donor‐acceptor distances in the previously characterized [Et_4_N]^+^I^−^⋅⋅⋅CBr_4_ complex [*d*(Br⋅⋅⋅I^−^)=3.30 Å].^[41]^ Similarly, the ca. 0.04 Å lengthening of the carbon‐bromine bond is observed due to transfer of electron density to the C−Br bond‐centered antibonding σ* orbital. Inspection of the spatial arrangement of the endohedral anions shows that the XB‐driven encapsulation of CBr_4_ has pushed the iodides significantly closer to the mesitylene core of **L** ^3+^, as the *d*(I^−^⋅⋅⋅Mes_centroid_) has decreased from 4.08 Å in the parent cage and 4.40 Å in the uncoordinated **L**(Tf_2_N)_2_^+^⋅⋅⋅I^−^ to 3.78–3.84 Å in the (CBr_4_⋅⋅⋅4 I^−^)@[**L**_4_(Cu_3_I_4_)_4_]^8+^ host–guest complex (Figure 3). This demonstrates the dynamic nature of the **L** ^3+^⋅⋅⋅I^−^ ionic bonds which hold the iodide anions in the cationic pocket. It is worth noting that on the basis of the crystallographic evidence, the formation of the host–guest complex appears to be driven solely by the CBr_4_⋅⋅⋅4 I^−^ halogen bonds provided by the endohedral iodide anions to form the observed onion‐like layered structure. On the other hand, the iodide anions of the Cu_3_I_4_^−^ clusters, which point toward the center of the cage lie clearly outside the van der Waals radii of the Br and I atoms [*d*(C−Br⋅⋅⋅I^−^_cluster_)=4.40–4.72 Å]. Fujita and co‐workers have earlier studied a molecular container consisting of encapsulated perfluoroalkyl iodide and pentafluorohalobenzene XB donors that are halogen bonded to NO_3_^−^ anions,[^42^, ^43^] while Rebek, Jr. and co‐workers have reported a host–guest system where halogen bonding occurs within in a hydrogen‐bonded capsule.^[44]^

Further exploration of the di‐, tri‐, and tetrahalomethane series, i.e. CH_4−*n*_X_*n*_, where 2≤*n*≤4 and X=Cl, Br, or I, as guests for [**L**_4_(Cu_3_I_4_)_4_]^8+^ led to the realization that, in addition to CBr_4_, only bromoform, CHBr_3_ can be trapped by the cage. The crystal structure of the bromoform host–guest complex is isomorphous with the CBr_4_ analogue. The Br⋅⋅⋅I^−^ distances are, however, ca. 0.1 Å shorter (3.15–3.19 Å) than in the CBr_4_ complex (Figure 3) in contrast to the expected XB donor strength of the CHBr_3_ versus CBr_4_ and also much shorter than the respective tetraalkylammonium iodide XB complexes.^[45]^ This is not due to stronger binding of CHBr_3_, but is most likely caused by the averaging of the respective C−Br⋅⋅⋅I^−^ and C−H⋅⋅⋅I^−^ distances, as indicated also by the slightly larger displacement ellipsoids of the CHBr_3_ guest compared to CBr_4_ (actual disorder of the CHBr_3_ was not detected). Calculation of interaction energies at the PBE0/def2‐TZVP level in acetonitrile (PCM) using experimental coordinates from the single‐crystal X‐ray structures estimates the (CBr_4_⋅⋅⋅4 I^−^)@[**L**_4_(Cu_3_I_4_)_4_]^8+^ complexation energy to be −23.9 kcal mol^−1^ with the (CHBr_3_⋅⋅⋅4 I^−^)@[**L**_4_(Cu_3_I_4_)_4_]^8+^ 10.8 kcal mol^−1^ less favorable. We suggest that the observed selectivity of [**L**_4_(Cu_3_I_4_)_4_]^8+^ toward CBr_4_ and CHBr_3_ arises from the size and shape complementarity as well as from the rather strong halogen bonds between the two bromomethanes and 4 I^−^@[**L**_4_(Cu_3_I_4_)_4_]^8+^ host. Therefore, iodomethanes, which are expected to be the strongest XB donors from this series are too large to form the endohedral CH_4−*n*_I_*n*_⋅⋅⋅4 I^−^ XB complex, as the estimated I^−^⋅⋅⋅Mes_centroid_ distance using CI_4_ or CHI_3_ as guests is between 3.2 and 3.3 Å. This is ca. 0.1–0.2 Å below the shortest known I^−^⋅⋅⋅C6_centroid_ distance and roughly 0.5 Å below the majority of known short I^−^⋅⋅⋅C6_centroid_ distances according to the CSD.

We also attempted to characterize the host–guest systems in solution, although the rather poor solubility of the parent cage hampers these analyses. The stepwise addition of CHBr_3_ into a 3 : 1 CD_3_CN/DMF‐d7 solution of host cage showed no shift of the CHBr_3_ proton signal. This is not necessarily indicative of weak binding, but may rather arise from the preferable solvation of bromoform in CD_3_CN/DMF‐d7 and thus results in a rapid exchange of guest on the NMR timescale. Similarly, introducing the bromoform guest into the copper(I) iodide reaction mixture before the assembly of the cage gave an identical CHBr_3_ proton shift compared to the free bromoform. However, the addition of a significant excess of CHBr_3_ results in the gradual disappearance of the signals corresponding to the empty cage and the appearance of a new set of signals arising from the methylene group next to the tertiary amine shifting downfield whereas the other signals are shifted upfield. This is accompanied by a yellow discoloration of the sample solution and a very small (ca. 0.02 ppm) upfield shift of the CHBr_3_ signal. Similar spectral changes of the host are also evident when a smaller excess of CBr_4_ is used as the guest (see Figure S7 as an example). After a week, dark brown crystals emerged from this yellow solution from which a preliminary crystal structure was obtained (Figure S8). The structure is a dinuclear cationic copper(II) complex [(CuI_3_)_2_ **L**]I, which suggests that, in the large presence of CBr_4_, a reaction similar to copper‐catalyzed bromination by CBr_4_ occurs^[46]^ and the cage disassembles. Furthermore, an incremental addition of CBr_4_ into a solution of the host cage results in a pale yellow coloration of the initially colorless solution, as signified by the appearance of two absorption bands at *λ*_max_=291 and 360 nm in the UV/Vis absorption spectrum of the cage, with the latter band close to the *λ*_max_=345 nm of CBr_4_⋅⋅⋅I^−^ complex reported earlier^[41]^ However, a control experiment carried out by measuring the UV/Vis spectrum of an acetonitrile solution containing iodine and tetrabutylammonium iodide confirms that the observed absorption bands in fact arise from the formation of triiodide in solution.

## Conclusion

In summary, we have demonstrated the assembly of a tetrahedral coordination cage from an uncommon Cu_3_I_4_^−^ cluster and cationic ligand **L** ^3+^. The step‐wise self‐assembly of the cage from copper(I) iodide and **L**(Tf_2_N)_3_ was monitored by ^1^H NMR spectroscopy, which revealed the initial formation of an **L** ^3+^⋅⋅⋅I^−^ ionic complex followed by the gradual formation of the [**L**_4_(Cu_3_I_4_)_4_]^8+^ cage, which encapsulates four iodide anions through **L** ^3+^⋅⋅⋅I^−^ interactions, as also evidenced by single‐crystal X‐ray diffraction studies. The interstitial space between these I^−^ anions was found to be suitable for selective halogen‐bond‐driven encapsulation of bromomethanes CBr_4_ and CHBr_3_, while smaller chloromethanes or larger iodomethanes were not accepted as guest species by the cage. Analysis of the solid‐state structures of the host–guest complexes led us to conclude that the selectivity is due to the size match of the (CHBr_3_⋅⋅⋅4 I^−^) and (CBr_4_⋅⋅⋅4 I^−^) with the endohedral space of the [**L**_4_(Cu_3_I_4_)_4_]^8+^ cage, while the potentially more stable halogen‐bond complexes (CHI_3_⋅⋅⋅4 I^−^) and (CI_4_⋅⋅⋅4 I^−^) are too large to fit within its cavity. The presented results show that cationic ligands can be useful in building onion‐like multilayer host–guest systems, which can be beneficial in terms of increased selectivity and binding strength. We are currently exploring the possibilities of extending this strategy beyond the presented guest species and cage topology.

## Conflict of interest

The authors declare no conflict of interest.

1

## Supporting information

As a service to our authors and readers, this journal provides supporting information supplied by the authors. Such materials are peer reviewed and may be re‐organized for online delivery, but are not copy‐edited or typeset. Technical support issues arising from supporting information (other than missing files) should be addressed to the authors.

Supporting Information

Supporting Information

Supporting Information

Supporting Information

Supporting Information

Supporting Information

## Data Availability

The data that support the findings of this study are available in the Supporting Information of this article.
