# Supplementary material for: Charge‐Assisted Halogen Bonding in an Ionic Cavity of a Coordination Cage Based on a Copper(I) Iodide Cluster
Source: Angew Chem Int Ed Engl. 2023 Jan 11;62(7):e202215689. doi: 10.1002/anie.202215689 (PMC10108208; doi:10.1002/anie.202215689)
Supplement: Supplementary file 1 — Supporting Information [file ANIE-62-0-s001.pdf]

Angewandte Chemie
Eine Zeitschrift der Gesellschaft Deutscher Chemiker

Supporting Information

Charge-Assisted Halogen Bonding in an Ionic Cavity of a Coordination Cage Based on a Copper(I) Iodide Cluster

A. Peuronen, A. I. Taponen, E. Kalenius, A. Lehtonen, M. Lahtinen**

Supporting Information

Contents

Experimental details 2
Synthesis 2
Methods 2
ESI-Q-TOF Mass spectrometry 5
XYZ data for DFT calculcations 8
References 14

Experimental details

All reagents and solvents were purchased from commercial sources and were used as received without further purification (solvents were dried over molecular sieves). Ligand **L**(Tf_2N)$_3$ was synthesized from tris(bromomethyl)mesitylene and an excess of DABCO following an anion exchange with lithium bis(trifluoromethanelsulfonyl)imide, $LiTf_2N$, according to the procedure described earlier.[1,2]

Synthesis

Typical procedure for the synthesis of $4I^-@[L_4(Cu_3I_4)_4]^{8+}$ cage: Solutions of 0.02 mmol (26.7 mg) of **L**(Tf_2N)$_3$ in 2 ml of MeCN and 0.12 mmol (22.9 mg) of copper(I) iodide in 3 ml of MeCN were combined and stirred for 15 min. The white precipitate that was formed was separated by centrifugation, washed three times with MeCN and dried in vacuo. Isolated yield 20.2 mg. ^{1}H NMR (500.06 MHz, CD_3CN/DMF-d7 (3:1), referenced to CD_3CN at 1.94 ppm, 25°C): δ = 4.78 (s, 24H), 3.66 (t, 72H), 3.40 (t, 72H), 2.59 (s, 36H). IR (ATR): 3485w(br), 2959m, 2887m, 2645vw, 2245w, 1614w, 1551w, 1487m, 1455s, 1404s, 1362vs, 1319s, 1240w, 1186m, 1135w, 1055vs, 1006s, 953s, 918vs, 882s, 854vs, 846vs, 798vs, 723w, 700w, 651w, 617vw, 576m, 549m, 531m, 509m, 423w, 409w cm^{-1}. Fluorescence (CH_3CN): λ_{ex} = 380 nm, λ_{em} = 539 nm

Host-guest crystallizations were conducted by *in situ* self-assembly of the host cage by mixing 5 µmol of **L**(Tf_2N)$_3$ dissolved in 1 ml of MeCN or DMF and 30 µmol of CuI in 3 ml of MeCN followed by the addition of the halomethane guest. Typical used host-guest ratio was 1:10, but higher amounts (up to 1000-fold excess) of guests were also attempted. The tested guest molecules were CH_2Cl_2, $CHCl_3$, CCl_4, CH_2Br_2, $CHBr_3$, CBr_4, CH_2I_2, CHI_3 and CI_4. Crystals were grown by either slow evaporation of the solvent, slow mixing of CuI and ligand solutions or by diffusion of diethyl ether into the reaction mixture in case MeCN/DMF was used as the reaction solvent. By this method crystals of host-guest complexes of only $CHBr_3$ and CBr_4 could be grown whereas the other halomethanes provided crystals of the $4I^-@[\mathbf{L}_4(Cu_3I_4)_4]^{8+}$ cage.

Methods

The **NMR spectra** were recorded on Bruker Avance III HD 300 (BBFO probe), Avance 500 (Smartprobe) and Avance 500 (Prodigy BBO CryoProbe) spectrometers. The reported signals are given as δ values in ppm using residual solvent signals as the internal standards. **IR spectra** were recorded using Bruker Alpha ATR (diamond) FT-IR spectrometer. **Fluorescence** spectra were measured with Edinburgh Instruments FLS1000 Photoluminescence spectrometer equipped with a xenon arc lamp excitation source and a PMT-900 detector.

Single crystal X-ray diffraction data were collected on Agilent/Rigaku dual-source (Cu/Mo) diffractometer equipped with multilayer optics for generation of monochromatized Cu K_α radiation and Atlas detector or Rigaku Oxford Diffraction custom system consisting of microfocus MicroMax™-007 HF rotating anode generator producing monochromatized Cu $K_{\alpha1}$ radiation and HyPix-6000HE detector (crystal data of $(CuI_3)_2$**L**I. Crystals were mounted on the instrument goniometer head using MiTeGen MicroMounts™ and Fomblin® oil. The data collection and reduction were carried out using CrysAlis^{Pro} software.[3] Crystal structures were solved and refined within the Olex^2 graphical interface[4] by using the SHELXS and SHELXL software, respectively.[5,6] All non-hydrogen atoms were refined using anisotropic displacement parameters (excluding structure of $(CuI_3)_2$**L**I) whereas H atoms were refined using a riding atom model. For cage crystal structures, PLATON SQUEEZE[7] was used to treat the unresolved electron density arising from disordered solvent molecules (and other potential atoms) that could not be located from the difference density map. These corresponded to unit cell volumes of ca. 30%, 15% and 37% for $4I^-@[\mathbf{L}_4(Cu_3I_4)_4]^{8+}$, $(CBr_4\cdots4I^-)@[\mathbf{L}_4(Cu_3I_4)_4]^{8+}$ and $(CHBr_3\cdots4I^-)@[\mathbf{L}_4(Cu_3I_4)_4]^{8+}$, respectively. Crystal structure of $4I^-@[\mathbf{L}_4(Cu_3I_4)_4]^{8+}$ consists of poorly resolved $Cu(I)_xI_y^{x-y}$ anions of which only ones residing in endohedral space of the cage could be satisfactorily refined. These endohedral anions appear to have CuI_3^{2-} structure and show four-fold disorder (0.125 occupancy) with one of the iodide arms possessing nearly identical fractional coordinated with the uncoordinated iodide atom (0.875 occupancy) that resides in the cationic pocket of $\mathbf{L}^{3+}$. The two bromomethane host-guest complexes, $(CBr_4\cdots4I^-)@[\mathbf{L}_4(Cu_3I_4)_4]^{8+}$ and $(CHBr_3\cdots4I^-)@[\mathbf{L}_4(Cu_3I_4)_4]^{8+}$, both appear to have a small amount of triiodide co-crystallized in their lattices, which is supported by the observation of formation of triiodide also in UV/vis studies.

Diffusion ordered NMR spectrum (DOSY) of cage was recorded by preparing a sample using CD_3CN/DMF-d7 (3:1) mixture of deuterated solvent (Figure S3). The viscosity of this mixture (molar fraction of acetonitrile is 0.816) was determined by fitting 2nd degree polynomial function (R^2 = 0.999) to the viscosity values (η) of different molar fractions of binary mixture of acetonitrile and DMF from ref.[8] This provided a value of $\eta = 4.137\text{x}10^{-4}$ Pa s, which was used together with the determined diffusion coefficient $D = 4.82 \times 10^{-10}$ $m^2 \cdot s^{-1}$ to estimate the van der Waals radius of the cage in solution as 1.10 nm by applying the Stokes-Einstein equation:

$$r = \frac{kT}{6\pi\eta D} \quad (1)$$

Computational analysis of the host-guest systems was carried out to determine approximate binding energies of the two guest molecules within the cage. All calculation were carried out using Gaussian16 program[9] using density functional theory (DFT) at the PBE0/def2-TZVP level (ECP on I atoms) together with polarizable continuum model (PCM) to take account solvent effects (acetonitrile). The binding energies were calculated as single point energies, *i.e.* $E_{Int} = E_{HG} - (E_{cage} + E_{guest})$, by using coordinates from single crystal X-ray data of the parent host cage and the respective $CHBr_3$ and CBr_4 host-guest complexes. Calculation of E_{cage} includes the atoms associated with the cage framework together with the four endohedral iodides that act as XB acceptors, whereas E_{guest} is calculated for the sole bromomethane guest. Atoms other than cage framework, endohedral iodides and guest molecules were excluded from the calculations. For comparison, we also calculated the binding energies of the respective halomethane···$4I^-$ systems. These were done by assuming the respective initial geometries on the basis of single crystal X-ray data of the host-guest complexes and carrying out geometry optimizations at the PBE0/def2-SVP level (PCM, acetonitrile) followed by frequency analyses. The binding energies were then calculated as single point calculations at the PBE0/def2-TZVP level (PCM, acetonitrile) using the optimized geometries.

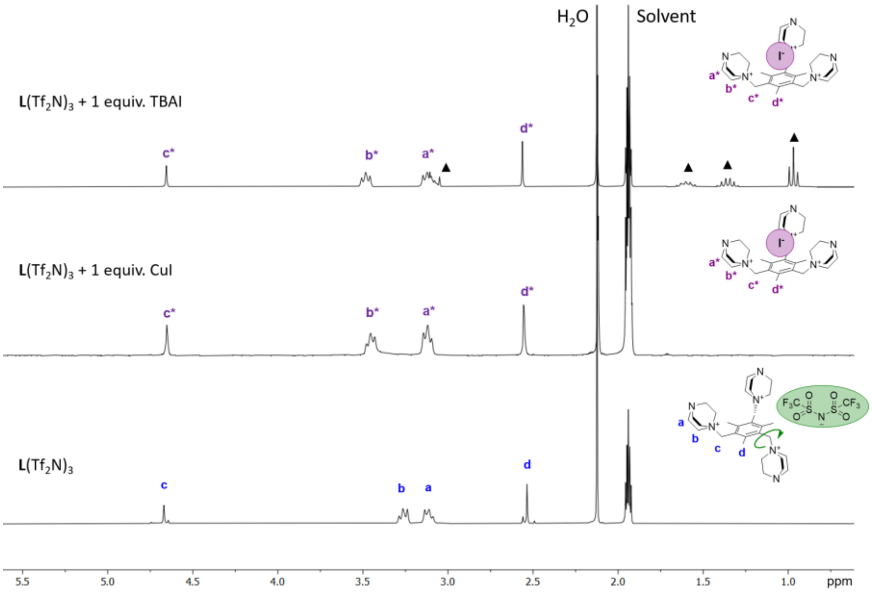

Figure S1. Comparison between the ^1H NMR spectra of **L**$(Tf_2N)_3$ (bottom) and **L**$(Tf_2N)_3$ after addition of copper(I) iodide (middle) and tetrabutylammonium iodide (top), respectively (300 MHz, 298 K, CD_3CN). ▲Peaks from tetrabutylammonium cation.

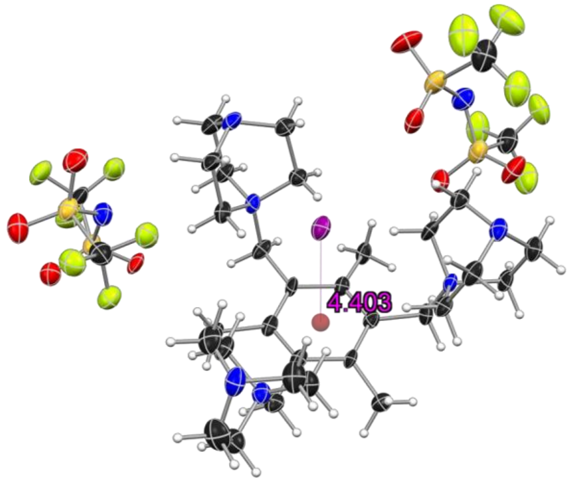

Figure S2. Illustration of the $\mathbf{L}^{3+}\cdots I^-$ unit present in the crystal structure of $L(Tf_2N)_2I$ obtained by 1:1 reaction between $L(Tf_2N)_3$ and tetrabutylammonium iodide. Disordered MeCN solvent molecules are omitted from the figure. Displacement ellipsoids are presented at the 50% probability level.

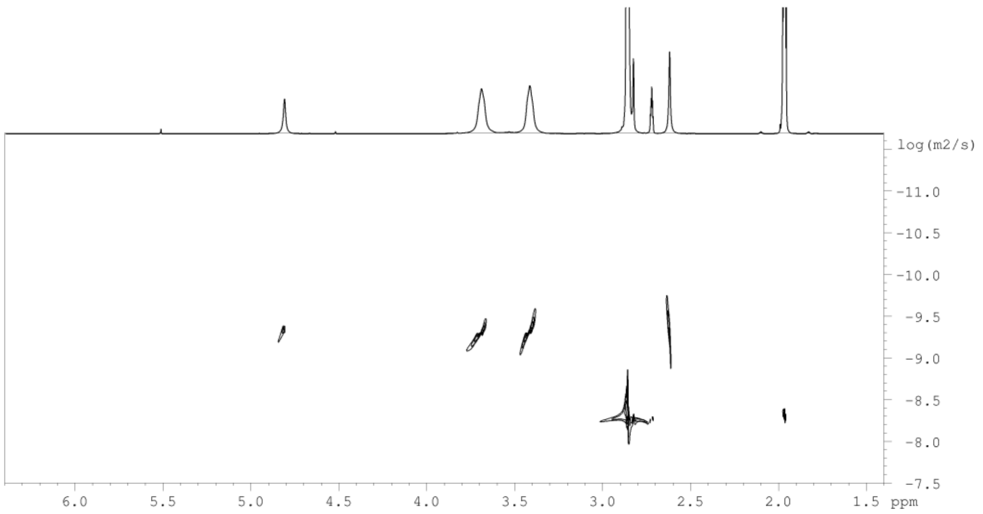

Figure S3. ^{1}H DOSY spectrum of $4I^-$@[$\mathbf{L}_4(Cu_3I_4)_4$]$^{8+}$ cage (500 MHz, 298K, 3:1 CD_3CN/DMF-d7).

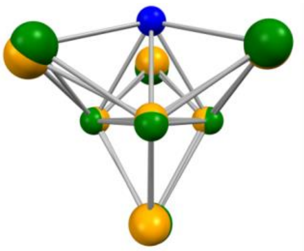

	Cu_4I_4 (CSD)	$Cu_3I_4^-$ in this work
d(Cu–I) / Å	2.70	2.66
d(Cu–Cu) / Å	2.71	2.57
d(I–I) / Å	4.49	4.47
∠(Cu_3-centroid–Cu–N) / °	159.3	163.9

Figure S4. Right: Comparison of SCXRD-derived geometric parameters between the $Cu_3I_4^-$, as determined using the crystal structure of the host in this work. and Cu_4I_4 clusters crystallized with N-donor ligands found in the Cambridge Structural Database (CSD). Left: Visual inspection of the similarity between the $Cu_3I_4^-$ (orange) and Cu_4I_4 (green) clusters with the additional Cu-atom of Cu_4I_4 highlighted in blue.

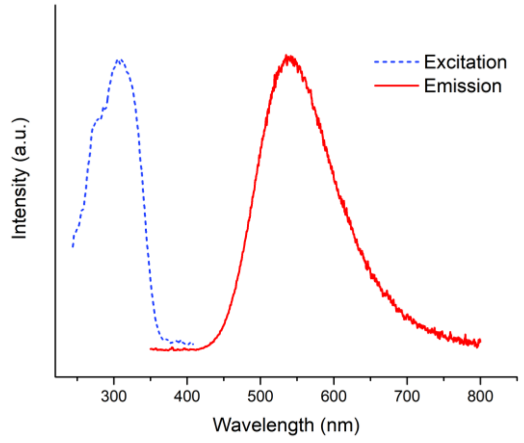

Figure S5. Excitation and emission spectra of bulk sample of the host cage.

Table S1. Calculated interaction energies (PBE0/def2-TZVP) of host-guest complexes of CBr_4 and $CHBr_3$ compared to the optimized geometries of respective halomethane-$4I^-$ systems.

XB system	E_{int} /kcal mol^{-1}
(CBr_4···$4I^-$)@[$\mathbf{L}_4(Cu_3I_4)_4$]$^{8+}$	-23.9
($CHBr_3$···$4I^-$)@[$\mathbf{L}_4(Cu_3I_4)_4$]$^{8+}$	-13.1
CBr_4···$4I^-$	-14.2
$CHBr_3$···$4I^-$	-11.3
CCl_4···$4I^-$	-7.5

ESI-Q-TOF Mass spectrometry

Mass spectrometric experiments were performed with ABSciex QSTAR Elite ESI-Q-TOF mass spectrometer, equipped with an API 200 TurboIonSpray ESI Source from AB Sciex. Solid sample was dissolved in 1 ml DMF, centrifuged, and further diluted in DMF (1:10). The *in situ* prepared solution of $L(Tf_2N)_3$, CuI and tetramethylammonium iodide in 1:3:3 ratio was diluted 1:3 in MeCN. Samples were injected with 5 µl flow rate to ESI source and spectra were externally calibrated using sodium trifluoroacetate. Experiments were performed on positive polarization and *m/z* range from 1200 to 2500. Parameters were optimized to get maximum abundance of ions under study. The measurements and data handling were accomplished with Analyst® QS 2.0 software. N_2 was used as drying and nebulization gas in all measurements.

Table S2. Ions observed in (+)ESI-MS analysis for solid cage dissolved in DMF, their accurate mass values, molecular weights and absolute mass accuracies (Δm/z).

Ion	Formula	*m/z*	Charge (+)	MW
$[L_4(Cu_3I_4)_4+I_5+(CuI)_3]^{3+}$	$C_{120}H_{204}N_{24}Cu_{15}I_{24}$	1993.927	3	5981.782
$[L_4(Cu_3I_4)_4+I_5+(CuI)_2]^{3+}$	$C_{120}H_{204}N_{24}Cu_{14}I_{23}$	1930.634	3	5791.902
$[L_4(Cu_3I_4)_4+I_5+CuI]^{3+}$	$C_{120}H_{204}N_{24}Cu_{13}I_{22}$	1866.850	3	5600.551
$[L_4(Cu_3I_4)_4+I_{4+}TFSI]^{3+}$	$C_{122}H_{204}O_4N_{25}S_2Cu_{12}F_6I_{20}$	1854.097	3	5562.291
$[L_4(Cu_3I_4)_4+I_5]^{3+}$	$C_{120}H_{204}N_{24}Cu_{12}I_{21}$	1803.335	3	5410.004
$[L_4(Cu_3I_4)_4+I_4]^{4+}$	$C_{120}H_{204}N_{24}Cu_{12}I_{20}$	1320.844	4	5283.377
$[L_4(Cu_3I_4)_3+I_6+(CuI)_2]^{3+}$	$C_{120}H_{204}N_{24}Cu_{11}I_{20}$	1740.145	3	5220.435
$[L_4(Cu_3I_4)_3+I_6+(CuI)]^{3+}$	$C_{120}H_{204}N_{24}Cu_{10}I_{19}$	1676.532	3	5029.595
$[L_3(Cu_3I_4)_4+(CuI)_2+2I]^{3+}$	$C_{90}H_{153}N_{18}Cu_{14}I_{20}$	1637.953	3	4913.859
$[L_3(Cu_3I_4)_4+CuI+2I]^{3+}$	$C_{90}H_{153}N_{18}Cu_{13}I_{19}$	1574.627	3	4723.881
$[L_3(Cu_3I_4)_4+2I]^{3+}$	$C_{90}H_{153}N_{18}Cu_{12}I_{18}$	1511.350	3	4534.049
$[L_3(Cu_3I_4)_3+(CuI)_2+3I]^{3+}$	$C_{90}H_{153}N_{18}Cu_{11}I_{17}$	1448.080	3	4344.241

Table S3. Ions observed in (+)ESI-MS analysis for *in situ* prepared sample, their accurate mass values, molecular weights and absolute mass accuracies (Δm/z).

Ion	Formula	m/z	Charge	MW
$[L_4(Cu_3I_4)_4+I_4+TFSI+CuI]^{3+}$	$C_{122}H_{204}O_4N_{25}S_2Cu_{13}F_6I_{21}$	1918.418	3	5755
$[L_4(Cu_3I_4)_4+I_5+CuI]^{3+}$	$C_{120}H_{204}N_{24}Cu_{13}I_{22}$	1867.187	3	5602
$[L_4(Cu_3I_4)_4+I_{4+}TFSI]^{3+}$	$C_{122}H_{204}O_4N_{25}S_2Cu_{12}F_6I_{20}$	1854.524	3	5564
$[L_4(Cu_3I_4)_4+I_5]^{3+}$	$C_{120}H_{204}N_{24}Cu_{12}I_{21}$	1803.529	3	5411
$[L_4(Cu_3I_4)_4+I_4]^{4+}$	$C_{120}H_{204}N_{24}Cu_{12}I_{20}$	1320.804	4	5283
$[L_3(Cu_3I_4)_4+(CuI)_3+I+TFSI]^{3+}$	$C_{92}H_{153}O_4N_{19}S_2Cu_{15}F_6I_{20}$	1752.709	3	5258
$[L_3(Cu_3I_4)_4+(CuI)_2+I+TFSI]^{3+}$	$C_{92}H_{153}O_4N_{19}Cu_{14}F_6I_{19}$	1689.399	3	5068
$[L_3(Cu_3I_4)_4+(CuI)_2+2I]^{3+}$	$C_{90}H_{153}N_{18}Cu_{14}I_{20}$	1637.867	3	4914
$[L_3(Cu_3I_4)_4+CuI+2I]^{3+}$	$C_{90}H_{153}N_{18}Cu_{13}I_{19}$	1574.601	3	4724
$[L_3(Cu_3I_4)_4+2I]^{3+}$	$C_{90}H_{153}N_{18}Cu_{12}I_{18}$	1511.640	3	4535
$[L_3(Cu_3I_4)_3+(CuI)_2+3I]^{3+}$	$C_{90}H_{153}N_{18}Cu_{11}I_{17}$	1448.025	3	4344

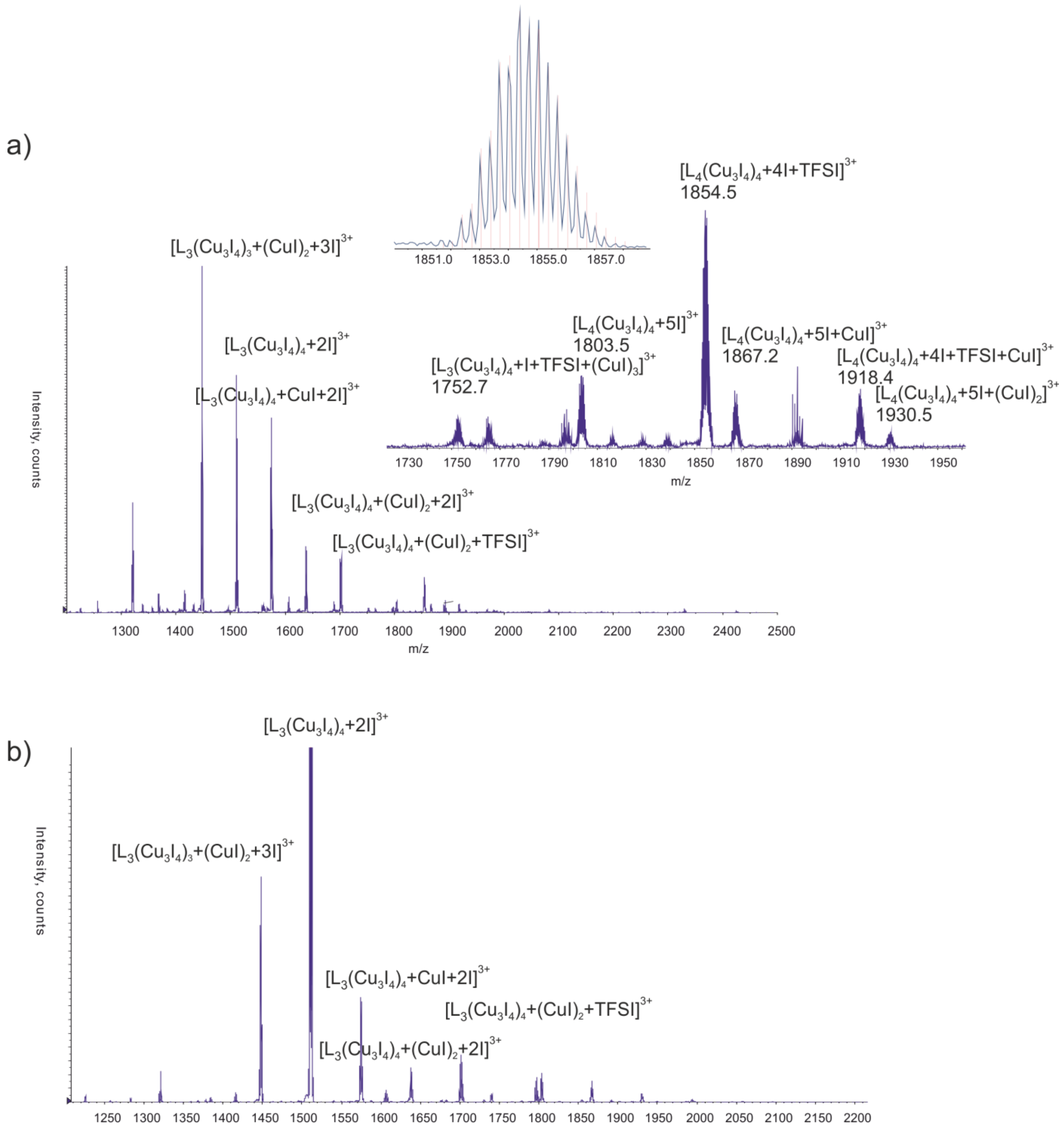

Figure S6. (+)ESI mass spectra measured from a) *in situ* prepared sample and b) solid sample dissolved in DMF. Insets show zoomed view for *m/z* range 1730 to 1950 and experimental and theoretical (red solid line) isotopic distribution for ion $[L_4(Cu_3I_4)_4+I_{4+}TFSI]^{3+}$.

Table S4. Crystallographic data for crystal structures presented in this work.

	[**L**$_4$(Cu$_3$I$_4$)$_4$]$^{8+}$	(CBr$_4$···4I$^-$)@[**L**$_4$(Cu$_3$I$_4$)$_4$]$^{8+}$	(CHBr$_3$···4I)@[**L**$_4$(Cu$_3$I$_4$)$_4$]$^{8+}$	**L**(Tf$_2$N)$_2$I	(CuI$_3$)$_2$-**L**I
CCDC deposition number	2211276	2211277	2211278	2211279	Preliminary structure
Empirical formula	$C_{120}H_{204}Cu_{13.5}I_{25.5}N_{24}$	$C_{145}H_{240}Br_4Cu_{14}I_{27.5}N_{36}$	$C_{123}H_{208}Br_3Cu_{13}I_{26}N_{25}$	$C_{35}H_{52.5}F_{12}IN_{8.5}O_8S_4$	$C_{39}H_{67}Cu_2I_7N_{10}O$
Formula weight	6076.8	7186.67	6402.28	1203.49	1707.4
Temperature/K	123.0(1)	123.0(1)	123.0(1)	120.0(1)	150.0(1)
Crystal system	tetragonal	tetragonal	tetragonal	monoclinic	orthorhombic
Space group	*I*-4	*P*-4	*P*-4	$P2_1/c$	*Pbca*
a/Å	21.2716(3)	21.23850(13)	21.3884(3)	9.0607(4)	16.2544(3)
b/Å	21.2716(3)	21.23850(13)	21.3884(3)	31.4196(13)	23.9236(5)
c/Å	28.4502(6)	27.7838(3)	28.2055(3)	16.7868(8)	25.4651(6)
α/°	90	90	90	90	90
β/°	90	90	90	99.461(4)	90
γ/°	90	90	90	90	90
Volume/Å^3	12873.2(5)	12532.6(2)	12903.0(4)	4713.9(4)	9902.5(4)
Z	2	2	2	4	8
ρ_{calc} g/cm^3	1.568	1.904	1.648	1.696	2.291
μ/mm^{-1}	25.429	28.924	26.354	7.946	35.662
F(000)	5670	6731	5962	2444	6464
Crystal size/mm^3	0.123 × 0.084 × 0.049	0.094 × 0.077 × 0.057	0.099 × 0.078 × 0.046	0.200 × 0.070 × 0.030	0.39 × 0.1 × 0.03
Radiation	Cu Kα (λ = 1.54184)	Cu Kα (λ = 1.54184)	Cu Kα (λ = 1.54184)	Cu Kα (λ = 1.54184)	Cu Kα (λ = 1.54184)
2Θ range for data collection/°	8.314 to 152.874	6.69 to 137.994	6.632 to 135.998	6.034 to 136.502	6.942 to 136.5
Index ranges	-26 ≤ h ≤ 25, -25 ≤ k ≤ 22, -34 ≤ l ≤ 34	-25 ≤ h ≤ 11, -19 ≤ k ≤ 24, -32 ≤ l ≤ 24	-22 ≤ h ≤ 25, -24 ≤ k ≤ 19, -33 ≤ l ≤ 19	-10 ≤ h ≤ 10, -36 ≤ k ≤ 37, -20 ≤ l ≤ 11	-7 ≤ h ≤ 19, -28 ≤ k ≤ 28, -30 ≤ l ≤ 30
Reflections collected	23238	22580	21914	16025	34013
Independent reflections	11257 [R_{int} = 0.0725, R_{sigma} = 0.0866]	17172 [R_{int} = 0.0276, R_{sigma} = 0.0490]	17987 [R_{int} = 0.0525, R_{sigma} = 0.0918]	8529 [R_{int} = 0.0789, R_{sigma} = 0.1001]	9026 [R_{int} = 0.0995, R_{sigma} = 0.0643]
Data/restraints/parameters	11257/13/448	17172/253/1053	17987/3/891	8529/12/636	9026/5/360
Goodness-of-fit on F^2	1.027	1.041	0.971	1.105	1.117
Final R indexes [I>=2σ (I)]	R_1 = 0.0781, wR_2 = 0.2089	R_1 = 0.0759, wR_2 = 0.2155	R_1 = 0.0655, wR_2 = 0.1728	R_1 = 0.1255, wR_2 = 0.3143	R_1 = 0.1251, wR_2 = 0.3609
Final R indexes [all data]	R_1 = 0.0918, wR_2 = 0.2276	R_1 = 0.0813, wR_2 = 0.2243	R_1 = 0.0924, wR_2 = 0.2024	R_1 = 0.1387, wR_2 = 0.3261	R_1 = 0.1306, wR_2 = 0.3823
Largest diff. peak/hole / e Å^{-3}	3.45/-1.72	4.18/-2.58	1.34/-1.48	5.37/-2.48	10.40/-2.69
Flack parameter	0.018(13)	0.152(8)	0.056(9)	-	-

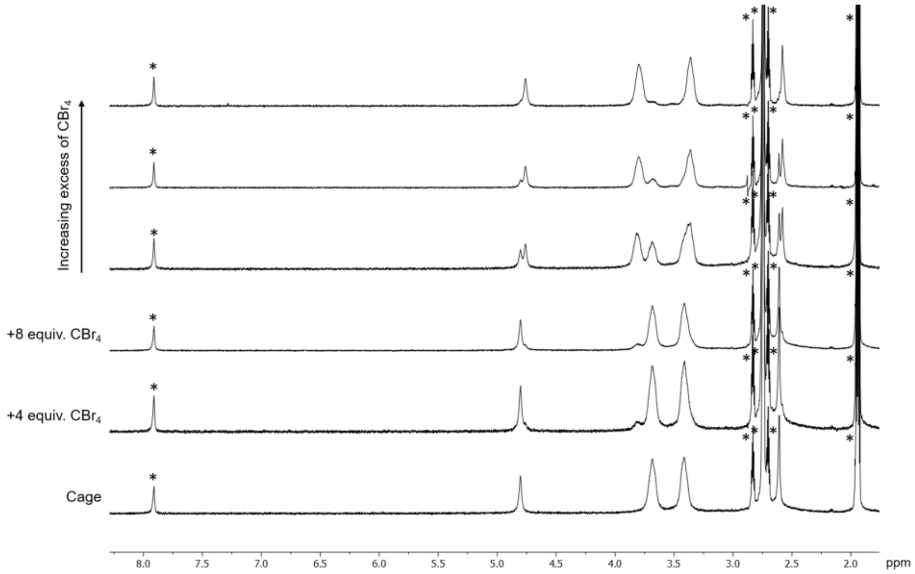

Figure S7. ^{1}H NMR spectra (500 MHz, 298 K) of empty cage (bottom) and incremental addition of CBr_4 in CD_3CN/DMF-d_7 (3:1). *solvent and H_2O peaks.

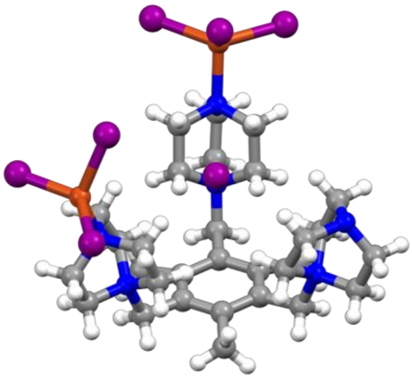

Figure S8. Partial crystal structure of $[(CuI_3)_2\mathbf{L}]I$ determined from crystals obtained after the addition of excess of CBr_4 into a solution of the cage compound.

XYZ data for DFT calculcations

<u>($CBr_4\cdots4I^-$)@$[L_4(Cu_3I_4)_4]^{8+}$ calculations</u>

($CBr_4\cdots4I^-$)@$[L_4(Cu_3I_4)_4]^{8+}$

C	-1.092280	-6.460758	-6.254130
C	-2.403349	-6.671639	-5.667892
C	-2.574966	-7.524218	-4.550983
C	-1.422467	-8.110893	-3.975859
C	-0.150353	-7.946767	-4.550983
C	0.000000	-7.038650	-5.673449
C	-3.626696	-6.048412	-6.373601
N	-4.026833	-4.652885	-5.901276
C	-5.223995	-4.258424	-6.748682
C	-5.573105	-2.800149	-6.376379
N	-4.849051	-2.356910	-5.198346
C	-5.099630	-3.294305	-4.189794
C	-4.446927	-4.654534	-4.481524
C	-2.922215	-3.629563	-6.037418
C	-3.367595	-2.305940	-5.417839
Cu	-5.551523	-0.433087	-4.670177
Cu	-4.881731	2.066859	-4.444018
Cu	-6.621739	0.969237	-2.896459
I	-3.986759	0.436200	-2.594728
C	-3.933818	-7.871650	-4.081436
C	-1.586410	-9.031036	-2.797826
N	-1.403163	-8.351389	-1.403079
C	-0.068763	-7.738140	-1.225263

C	0.106999	-7.270586	0.222272
N	-1.155405	-7.365041	0.944652
C	-2.177960	-6.660389	0.172263
C	-2.468474	-7.285590	-1.191921
C	-1.566740	-9.432859	-0.388969
C	-1.595379	-8.811596	0.975215
Cu	-0.969237	-6.621739	2.896459
Cu	-2.066859	-4.881731	4.444018
Cu	0.433087	-5.551523	4.670177
N	2.356910	-4.849051	5.198346
C	2.800149	-5.573105	6.376379
C	4.258424	-5.223995	6.748682
N	4.652885	-4.026833	5.901276
C	4.654534	-4.446927	4.481524
C	3.294305	-5.099630	4.189794
C	3.629563	-2.922215	6.037418
C	2.305940	-3.367595	5.417839
C	6.048412	-3.626696	6.373601
C	6.671639	-2.403349	5.667892
C	7.524218	-2.574966	4.550983
C	8.110893	-1.422467	3.975859
C	7.946767	-0.150353	4.550983
C	7.038650	0.000000	5.673449
C	6.460758	-1.092280	6.254130
C	7.871650	-3.933818	4.081436
C	9.031036	-1.586410	2.797826
N	8.351389	-1.403163	1.403079

C	7.738140	-0.068763	1.225263
C	7.270586	0.106999	-0.222272
N	7.365041	-1.155405	-0.944652
C	6.660389	-2.177960	-0.172263
C	7.285590	-2.468474	1.191921
C	9.432859	-1.566740	0.388969
C	8.811596	-1.595379	-0.975215
Cu	6.621739	-0.969237	-2.896459
Cu	4.881731	-2.066859	-4.444018
N	3.615733	-3.693912	-4.912176
C	2.263837	-3.174485	-5.306705
C	1.434808	-4.365194	-5.904056
N	2.146809	-5.636767	-5.712347
C	2.485484	-5.832308	-4.273146
C	3.394361	-4.576914	-3.870282
C	3.441616	-5.591309	-6.479180
C	4.261283	-4.340507	-6.062425
C	8.778111	0.957098	4.089772
C	6.869129	1.333731	6.265244
N	5.636767	2.146809	5.712347
C	4.365194	1.434808	5.904056
C	3.174485	2.263837	5.306705
N	3.693912	3.615733	4.912176
C	4.576914	3.394361	3.870282
C	5.832308	2.485484	4.273146
C	5.591309	3.441616	6.479180
C	4.340507	4.261283	6.062425

Cu	2.066859	4.881731	4.444018
Cu	0.969237	6.621739	2.896459
N	1.155405	7.365041	0.944652
C	-0.106999	7.270586	0.222272
C	0.068763	7.738140	-1.225263
N	1.403163	8.351389	-1.403079
C	2.468474	7.285590	-1.191921
C	2.177960	6.660389	0.172263
C	1.566740	9.432859	-0.388969
C	1.595379	8.811596	0.975215
C	5.659444	-1.001095	7.551635
C	1.586410	9.031036	-2.797826
C	1.422467	8.110893	-3.975859
C	2.574966	7.524218	-4.550983
C	2.403349	6.671639	-5.667892
C	1.092280	6.460758	-6.254130
C	-0.000000	7.038650	-5.673449
C	0.150353	7.946767	-4.550983
C	3.626696	6.048412	-6.373601
N	4.026833	4.652885	-5.901276
C	5.223995	4.258424	-6.748682
C	5.573105	2.800149	-6.376379
N	4.849051	2.356910	-5.198346
C	5.099630	3.294305	-4.189794
C	4.446927	4.654534	-4.481524
C	2.922215	3.629563	-6.037418
C	3.367595	2.305940	-5.417839
Cu	5.551523	0.433087	-4.670177
I	3.986759	-0.436200	-2.594728
C	3.933818	7.871650	-4.081436
C	-0.957098	8.778111	-4.089772
C	-1.333731	6.869129	-6.265244
N	-2.146809	5.636767	-5.712347
C	-1.434808	4.365194	-5.904056
C	-2.263837	3.174485	-5.306705
N	-3.615733	3.693912	-4.912176
C	-3.394361	4.576914	-3.870282
C	-2.485484	5.832308	-4.273146
C	-3.441616	5.591309	-6.479180
C	-4.261283	4.340507	-6.062425
C	1.001095	5.659444	-7.551635
C	1.333731	-6.869129	-6.265244
C	0.957098	-8.778111	-4.089772
C	-1.001095	-5.659444	-7.551635
I	-0.803234	8.086989	4.124502
Cu	-0.433087	5.551523	4.670177
N	-2.356910	4.849051	5.198346
C	-2.800149	5.573105	6.376379
C	-4.258424	5.223995	6.748682
N	-4.652885	4.026833	5.901276
C	-4.654534	4.446927	4.481524
C	-3.294305	5.099630	4.189794
C	-3.629563	2.922215	6.037418
C	-2.305940	3.367595	5.417839
C	-6.048412	3.626696	6.373601
C	-6.671639	2.403349	5.667892
C	-7.524218	2.574966	4.550983
C	-8.110893	1.422467	3.975859
C	-7.946767	0.150353	4.550983
C	-7.038650	-0.000000	5.673449
C	-6.460758	1.092280	6.254130
C	-7.871650	3.933818	4.081436
C	-9.031036	1.586410	2.797826
N	-8.351389	1.403163	1.403079
C	-7.738140	0.068763	1.225263
C	-7.270586	-0.106999	-0.222272
N	-7.365041	1.155405	-0.944652
C	-6.660389	2.177960	-0.172263
C	-7.285590	2.468474	1.191921
C	-9.432859	1.566740	0.388969
C	-8.811596	1.595379	-0.975215
C	-8.778111	-0.957098	4.089772
C	-6.869129	-1.333731	6.265244
N	-5.636767	-2.146809	5.712347
C	-4.365194	-1.434808	5.904056
C	-3.174485	-2.263837	5.306705
N	-3.693912	-3.615733	4.912176
C	-4.576914	-3.394361	3.870282
C	-5.832308	-2.485484	4.273146
C	-5.591309	-3.441616	6.479180
C	-4.340507	-4.261283	6.062425
C	-5.659444	1.001095	7.551635
I	0.819474	4.802118	6.685893
I	0.436200	3.986759	2.594728
I	3.332279	6.982500	3.729693
I	-0.819474	-4.802118	6.685893
I	0.803234	-8.086989	4.124502
I	-0.436200	-3.986759	2.594728
I	-3.332279	-6.982500	3.729693
I	4.802118	-0.819474	-6.685893

I	8.086989	0.803234	-4.124502
I	6.982500	-3.332279	-3.729693
I	-4.802118	0.819474	-6.685893
I	-8.086989	-0.803234	-4.124502
I	-6.982500	3.332279	-3.729693
Br	-0.233813	-1.585263	-1.122466
C	0.000000	0.000000	0.000000
Br	0.233813	1.585263	-1.122466
Br	-1.585263	0.233813	1.122466
Br	1.585263	-0.233813	1.122466
I	-0.797740	-4.286877	-2.855340
I	-4.286877	0.797740	2.855340
I	4.286877	-0.797740	2.855340
I	0.797740	4.286877	-2.855340
H	-2.729502	6.545198	6.206512
H	-2.206048	5.352190	7.137380
H	-4.325058	5.000261	7.711589
H	-4.856029	5.990102	6.561090
H	-2.945989	4.753293	3.330945
H	-3.416766	6.076904	4.097555
H	-5.385681	5.093225	4.317715
H	-4.790311	3.662490	3.893068
H	-1.555805	3.139489	6.023084
H	-2.163025	2.901861	4.555932
H	-3.955887	2.106574	5.581961
H	-3.496005	2.708769	6.995573
H	-6.652453	4.400051	6.249079
H	-6.005246	3.436340	7.344234
H	-6.333554	-0.425534	-0.231740
H	-7.831719	-0.788302	-0.671335
H	-8.398321	-0.634022	1.448236
H	-6.966148	-0.027140	1.836875
H	-6.643506	3.017055	-0.698536
H	-5.724556	1.888279	-0.038560
H	-6.599409	2.419631	1.903611
H	-7.690539	3.371342	1.202487
H	-8.864038	2.515978	-1.336786
H	-9.321575	1.000955	-1.578558
H	-9.926559	2.409224	0.554152
H	-10.073475	0.813863	0.452436
H	-9.431744	2.490380	2.837008
H	-9.766277	0.929277	2.880267
H	-2.800606	-1.801187	4.517012
H	-2.456361	-2.361422	5.982051
H	-4.213695	-1.285152	6.871743
H	-4.410136	-0.551365	5.460243
H	-4.906454	-4.268495	3.541663
H	-4.089877	-2.957771	3.128322
H	-5.858704	-1.664143	3.721339
H	-6.678807	-2.983360	4.142904
H	-3.703682	-4.311239	6.819844
H	-4.607495	-5.184152	5.822323
H	-6.410404	-3.967525	6.296343
H	-5.557545	-3.252167	7.450676
H	-7.692994	-1.858163	6.099855
H	-6.768426	-1.235176	7.243770
H	-7.343839	4.150891	3.285354
H	-7.677455	4.583339	4.789623
H	-8.826316	3.969174	3.860118
H	-9.609069	-0.605247	3.709171
H	-8.987185	-1.544928	4.845529
H	-8.294737	-1.466109	3.404938
H	-4.814504	0.533343	7.381992
H	-6.176940	0.506815	8.221119
H	-5.472027	1.903371	7.883712
H	2.729502	-6.545198	6.206512
H	2.206048	-5.352190	7.137380
H	4.325058	-5.000261	7.711589
H	4.856029	-5.990102	6.561090
H	2.945989	-4.753293	3.330945
H	3.416766	-6.076904	4.097555
H	5.385681	-5.093225	4.317715
H	4.790311	-3.662490	3.893068
H	1.555805	-3.139489	6.023084
H	2.163025	-2.901861	4.555932
H	3.955887	-2.106574	5.581961
H	3.496005	-2.708769	6.995573
H	6.652453	-4.400051	6.249079
H	6.005246	-3.436340	7.344234
H	6.333554	0.425534	-0.231740
H	7.831719	0.788302	-0.671335
H	8.398321	0.634022	1.448236
H	6.966148	0.027140	1.836875
H	6.643506	-3.017055	-0.698536
H	5.724556	-1.888279	-0.038560
H	6.599409	-2.419631	1.903611
H	7.690539	-3.371342	1.202487
H	8.864038	-2.515978	-1.336786
H	9.321575	-1.000955	-1.578558
H	9.926559	-2.409224	0.554152

H	10.073475	-0.813863	0.452436
H	9.431744	-2.490380	2.837008
H	9.766277	-0.929277	2.880267
H	2.800606	1.801187	4.517012
H	2.456361	2.361422	5.982051
H	4.213695	1.285152	6.871743
H	4.410136	0.551365	5.460243
H	4.906454	4.268495	3.541663
H	4.089877	2.957771	3.128322
H	5.858704	1.664143	3.721339
H	6.678807	2.983360	4.142904
H	3.703682	4.311239	6.819844
H	4.607495	5.184152	5.822323
H	6.410404	3.967525	6.296343
H	5.557545	3.252167	7.450676
H	7.692994	1.858163	6.099855
H	6.768426	1.235176	7.243770
H	7.343839	-4.150891	3.285354
H	7.677455	-4.583339	4.789623
H	8.826316	-3.969174	3.860118
H	9.609069	0.605247	3.709171
H	8.987185	1.544928	4.845529
H	8.294737	1.466109	3.404938
H	4.814504	-0.533343	7.381992
H	6.176940	-0.506815	8.221119
H	5.472027	-1.903371	7.883712
H	6.545198	2.729502	-6.206512
H	5.352190	2.206048	-7.137380
H	5.000261	4.325058	-7.711589
H	5.990102	4.856029	-6.561090
H	4.753293	2.945989	-3.330945
H	6.076904	3.416766	-4.097555
H	5.093225	5.385681	-4.317715
H	3.662490	4.790311	-3.893068
H	3.139489	1.555805	-6.023084
H	2.901861	2.163025	-4.555932
H	2.106574	3.955887	-5.581961
H	2.708769	3.496005	-6.995573
H	4.400051	6.652453	-6.249079
H	3.436340	6.005246	-7.344234
H	-0.425534	6.333554	0.231740
H	-0.788302	7.831719	0.671335
H	-0.634022	8.398321	-1.448236
H	-0.027140	6.966148	-1.836875
H	3.017055	6.643506	0.698536
H	1.888279	5.724556	0.038560
H	2.419631	6.599409	-1.903611
H	3.371342	7.690539	-1.202487
H	2.515978	8.864038	1.336786
H	1.000955	9.321575	1.578558
H	2.409224	9.926559	-0.554152
H	0.813863	10.073475	-0.452436
H	2.490380	9.431744	-2.837008
H	0.929277	9.766277	-2.880267
H	-1.801187	2.800606	-4.517012
H	-2.361422	2.456361	-5.982051
H	-1.285152	4.213695	-6.871743
H	-0.551365	4.410136	-5.460243
H	-4.268495	4.906454	-3.541663
H	-2.957771	4.089877	-3.128322
H	-1.664143	5.858704	-3.721339
H	-2.983360	6.678807	-4.142904
H	-4.311239	3.703682	-6.819844
H	-5.184152	4.607495	-5.822323
H	-3.967525	6.410404	-6.296343
H	-3.252167	5.557545	-7.450676
H	-1.858163	7.692994	-6.099855
H	-1.235176	6.768426	-7.243770
H	4.150891	7.343839	-3.285354
H	4.583339	7.677455	-4.789623
H	3.969174	8.826316	-3.860118
H	-0.605247	9.609069	-3.709171
H	-1.544928	8.987185	-4.845529
H	-1.466109	8.294737	-3.404938
H	0.533343	4.814504	-7.381992
H	0.506815	6.176940	-8.221119
H	1.903371	5.472027	-7.883712
H	-6.545198	-2.729502	-6.206512
H	-5.352190	-2.206048	-7.137380
H	-5.000261	-4.325058	-7.711589
H	-5.990102	-4.856029	-6.561090
H	-4.753293	-2.945989	-3.330945
H	-6.076904	-3.416766	-4.097555
H	-5.093225	-5.385681	-4.317715
H	-3.662490	-4.790311	-3.893068
H	-3.139489	-1.555805	-6.023084
H	-2.901861	-2.163025	-4.555932
H	-2.106574	-3.955887	-5.581961
H	-2.708769	-3.496005	-6.995573
H	-4.400051	-6.652453	-6.249079

H	-3.436340	-6.005246	-7.344234
H	0.425534	-6.333554	0.231740
H	0.788302	-7.831719	0.671335
H	0.634022	-8.398321	-1.448236
H	0.027140	-6.966148	-1.836875
H	-3.017055	-6.643506	0.698536
H	-1.888279	-5.724556	0.038560
H	-2.419631	-6.599409	-1.903611
H	-3.371342	-7.690539	-1.202487
H	-2.515978	-8.864038	1.336786
H	-1.000955	-9.321575	1.578558
H	-2.409224	-9.926559	-0.554152
H	-0.813863	-10.073475	-0.452436
H	-2.490380	-9.431744	-2.837008
H	-0.929277	-9.766277	-2.880267
H	1.801187	-2.800606	-4.517012
H	2.361422	-2.456361	-5.982051
H	1.285152	-4.213695	-6.871743
H	0.551365	-4.410136	-5.460243
H	4.268495	-4.906454	-3.541663
H	2.957771	-4.089877	-3.128322
H	1.664143	-5.858704	-3.721339
H	2.983360	-6.678807	-4.142904
H	4.311239	-3.703682	-6.819844
H	5.184152	-4.607495	-5.822323
H	3.967525	-6.410404	-6.296343
H	3.252167	-5.557545	-7.450676
H	1.858163	-7.692994	-6.099855
H	1.235176	-6.768426	-7.243770
H	-4.150891	-7.343839	-3.285354
H	-4.583339	-7.677455	-4.789623
H	-3.969174	-8.826316	-3.860118
H	0.605247	-9.609069	-3.709171
H	1.544928	-8.987185	-4.845529
H	1.466109	-8.294737	-3.404938
H	-0.533343	-4.814504	-7.381992
H	-0.506815	-6.176940	-8.221119
H	-1.903371	-5.472027	-7.883712

4I$^-$@[$L_4(Cu_3I_4)_4$]$^{8+}$

C	1.158359	6.449239	-6.254130
C	2.471518	6.646688	-5.667892
C	2.651852	7.497465	-4.550983
C	1.505420	8.095907	-3.975859
C	0.231692	7.944812	-4.550983
C	0.072051	7.038282	-5.673449
C	3.688421	6.010971	-6.373601
N	4.074252	4.611420	-5.901276
C	5.267312	4.204726	-6.748682
C	5.601477	2.742954	-6.376379
N	4.872923	2.307149	-5.198346
C	5.133085	3.241930	-4.189794
C	4.494340	4.608769	-4.481524
C	2.959216	3.599459	-6.037418
C	3.391023	2.271347	-5.417839
Cu	5.555666	0.376236	-4.670177
Cu	4.860318	-2.116723	-4.444019
Cu	6.611471	-1.036969	-2.896459
I	3.982085	-0.476988	-2.594728
C	4.014190	7.830970	-4.081436
C	1.678774	9.014323	-2.797826
N	1.488578	8.336588	-1.403079
C	0.147971	7.737030	-1.225263
C	-0.032568	7.271300	0.222272
N	1.230737	7.352828	0.944652
C	2.246025	6.637745	0.172263
C	2.542924	7.259939	-1.191921
C	1.663217	9.416326	-0.388969
C	1.685495	8.794804	0.975215
Cu	1.036969	6.611471	2.896459
Cu	2.116723	4.860318	4.444019
Cu	-0.376236	5.555666	4.670177
N	-2.307149	4.872923	5.198346
C	-2.742954	5.601477	6.376379
C	-4.204726	5.267312	6.748682
N	-4.611420	4.074252	5.901276
C	-4.608769	4.494340	4.481524
C	-3.241930	5.133085	4.189794
C	-3.599459	2.959216	6.037418
C	-2.271347	3.391023	5.417839
C	-6.010971	3.688421	6.373601
C	-6.646688	2.471518	5.667892
C	-7.497465	2.651852	4.550983
C	-8.095907	1.505420	3.975859
C	-7.944812	0.231692	4.550983
C	-7.038282	0.072051	5.673449
C	-6.449239	1.158359	6.254130
C	-7.830970	4.014190	4.081436
C	-9.014323	1.678774	2.797826
N	-8.336588	1.488578	1.403079
C	-7.737030	0.147971	1.225263
C	-7.271300	-0.032568	-0.222272
N	-7.352828	1.230737	-0.944652
C	-6.637745	2.246025	-0.172263
C	-7.259939	2.542924	1.191921
C	-9.416326	1.663217	0.388969
C	-8.794804	1.685495	-0.975215
Cu	-6.611471	1.036969	-2.896459
Cu	-4.860318	2.116723	-4.444019
N	-3.577731	3.730731	-4.912176
C	-2.231222	3.197492	-5.306705
C	-1.390048	4.379652	-5.904056
N	-2.088996	5.658448	-5.712347
C	-2.425651	5.857445	-4.273146
C	-3.347332	4.611420	-3.870282
C	-3.384200	5.626246	-6.479180
C	-4.216628	4.383900	-6.062425
C	-8.787449	-0.867191	4.089772
C	-6.882422	-1.263345	6.265244
N	-5.658448	-2.088996	5.712347
C	-4.379652	-1.390048	5.904056
C	-3.197492	-2.231222	5.306705
N	-3.730731	-3.577731	4.912176
C	-4.611420	-3.347332	3.870282
C	-5.857445	-2.425651	4.273146
C	-5.626246	-3.384200	6.479180
C	-4.383900	-4.216628	6.062425
Cu	-2.116723	-4.860318	4.444019
Cu	-1.036969	-6.611471	2.896459
N	-1.230737	-7.352828	0.944652
C	0.032568	-7.271300	0.222272
C	-0.147971	-7.737030	-1.225263
N	-1.488578	-8.336588	-1.403079
C	-2.542924	-7.259939	-1.191921
C	-2.246025	-6.637745	0.172263
C	-1.663217	-9.416326	-0.388969
C	-1.685495	-8.794804	0.975215
C	-5.648900	1.058976	7.551635
C	-1.678774	-9.014323	-2.797826
C	-1.505420	-8.095907	-3.975859
C	-2.651852	-7.497465	-4.550983
C	-2.471518	-6.646688	-5.667892
C	-1.158359	-6.449239	-6.254130
C	-0.072051	-7.038282	-5.673449
C	-0.231692	-7.944812	-4.550983
C	-3.688421	-6.010971	-6.373601
N	-4.074252	-4.611420	-5.901276
C	-5.267312	-4.204726	-6.748682
C	-5.601477	-2.742954	-6.376379
N	-4.872923	-2.307149	-5.198346
C	-5.133085	-3.241930	-4.189794
C	-4.494340	-4.608769	-4.481524
C	-2.959216	-3.599459	-6.037418
C	-3.391023	-2.271347	-5.417839
Cu	-5.555666	-0.376236	-4.670177
I	-3.982085	0.476988	-2.594728
C	-4.014190	-7.830970	-4.081436
C	0.867191	-8.787449	-4.089772
C	1.263345	-6.882422	-6.265244
N	2.088996	-5.658448	-5.712347
C	1.390048	-4.379652	-5.904056
C	2.231222	-3.197492	-5.306705
N	3.577731	-3.730731	-4.912176
C	3.347332	-4.611420	-3.870282
C	2.425651	-5.857445	-4.273146
C	3.384200	-5.626246	-6.479180
C	4.216628	-4.383900	-6.062425
C	-1.058976	-5.648900	-7.551635
C	-1.263345	6.882422	-6.265244
C	-0.867191	8.787449	-4.089772
C	1.058976	5.648900	-7.551635
I	0.720409	-8.094787	4.124502
Cu	0.376236	-5.555666	4.670177
N	2.307149	-4.872923	5.198346
C	2.742954	-5.601477	6.376379
C	4.204726	-5.267312	6.748682
N	4.611420	-4.074252	5.901276
C	4.608769	-4.494340	4.481524
C	3.241930	-5.133085	4.189794
C	3.599459	-2.959216	6.037418
C	2.271347	-3.391023	5.417839
C	6.010971	-3.688421	6.373601
C	6.646688	-2.471518	5.667892
C	7.497465	-2.651852	4.550983
C	8.095907	-1.505420	3.975859
C	7.944812	-0.231692	4.550983
C	7.038282	-0.072051	5.673449
C	6.449239	-1.158359	6.254130
C	7.830970	-4.014190	4.081436
C	9.014323	-1.678774	2.797826
N	8.336588	-1.488578	1.403079
C	7.737030	-0.147971	1.225263
C	7.271300	0.032568	-0.222272
N	7.352828	-1.230737	-0.944652
C	6.637745	-2.246025	-0.172263
C	7.259939	-2.542924	1.191921
C	9.416326	-1.663217	0.388969
C	8.794804	-1.685495	-0.975215
C	8.787449	0.867191	4.089772
C	6.882422	1.263345	6.265244
N	5.658448	2.088996	5.712347
C	4.379652	1.390048	5.904056
C	3.197492	2.231222	5.306705
N	3.730731	3.577731	4.912176
C	4.611420	3.347332	3.870282
C	5.857445	2.425651	4.273146
C	5.626246	3.384200	6.479180
C	4.383900	4.216628	6.062425
C	5.648900	-1.058976	7.551635
I	-0.868588	-4.793478	6.685893
I	-0.476988	-3.982085	2.594728
I	-3.403581	-6.948023	3.729693
I	0.868588	4.793478	6.685893
I	-0.720409	8.094787	4.124502
I	0.476988	3.982085	2.594728
I	3.403581	6.948023	3.729693
I	-4.793478	0.868588	-6.685893
I	-8.094787	-0.720409	-4.124502
I	-6.948023	3.403581	-3.729693
I	4.793478	-0.868588	-6.685893
I	8.094787	0.720409	-4.124502
I	6.948023	-3.403581	-3.729693
I	0.841581	4.278486	-2.855340
I	4.278486	-0.841581	2.855340
I	-4.278486	0.841581	2.855340
I	-0.841581	-4.278486	-2.855340
H	2.662359	-6.572796	6.206512
H	2.151145	-5.374491	7.137380
H	4.273646	-5.044273	7.711589
H	4.794457	-6.039497	6.561090
H	2.897178	-4.783200	3.330945
H	3.354381	-6.111561	4.097555
H	5.333262	-5.148088	4.317715
H	4.752569	-3.711334	3.893068
H	1.523586	-3.155251	6.023083
H	2.133207	-2.923851	4.555932
H	3.934116	-2.146958	5.581961
H	3.468094	-2.744414	6.995573
H	6.607063	-4.467919	6.249079
H	5.969755	-3.497632	7.344233
H	6.337578	0.360678	-0.231740
H	7.839378	0.708091	-0.671335
H	8.404371	0.548019	1.448236
H	6.966061	-0.044171	1.836875
H	6.612274	-3.084903	-0.698536
H	5.704927	-1.946780	-0.038560
H	6.574294	-2.487059	1.903611
H	7.655625	-3.449890	1.202487
H	8.837819	-2.606583	-1.336786
H	9.310840	-1.096322	-1.578558
H	9.901377	-2.510711	0.554152
H	10.064616	-0.916938	0.452436
H	9.405757	-2.586797	2.837008
H	9.756252	-1.029201	2.880267
H	2.818897	1.772424	4.517012
H	2.480405	2.336154	5.982051
H	4.226630	1.241951	6.871743
H	4.415549	0.506192	5.460243
H	4.949892	4.218046	3.541663
H	4.119940	2.915750	3.128322
H	5.875432	1.604083	3.721339
H	6.708996	2.914836	4.142905
H	3.747620	4.273101	6.819844
H	4.660321	5.136716	5.822323
H	6.450682	3.901697	6.296343
H	5.590545	3.195106	7.450676
H	7.711612	1.779316	6.099855
H	6.780715	1.165826	7.243770
H	7.300964	-4.225848	3.285354
H	7.630135	-4.661689	4.789623
H	8.785223	-4.059317	3.860118
H	9.614761	0.506852	3.709171
H	9.002529	1.452849	4.845529
H	8.309310	1.381123	3.404938
H	4.808792	-0.582599	7.381992
H	6.171429	-0.570019	8.221119
H	5.452256	-1.959286	7.883712

H	-2.662359	6.572796	6.206512
H	-2.151145	5.374491	7.137380
H	-4.273646	5.044273	7.711589
H	-4.794457	6.039497	6.561090
H	-2.897178	4.783200	3.330945
H	-3.354381	6.111561	4.097555
H	-5.333262	5.148088	4.317715
H	-4.752569	3.711334	3.893068
H	-1.523586	3.155251	6.023083
H	-2.133207	2.923851	4.555932
H	-3.934116	2.146958	5.581961
H	-3.468094	2.744414	6.995573
H	-6.607063	4.467919	6.249079
H	-5.969755	3.497632	7.344233
H	-6.337578	-0.360678	-0.231740
H	-7.839378	-0.708091	-0.671335
H	-8.404371	-0.548019	1.448236
H	-6.966061	0.044171	1.836875
H	-6.612274	3.084903	-0.698536
H	-5.704927	1.946780	-0.038560
H	-6.574294	2.487059	1.903611
H	-7.655625	3.449890	1.202487
H	-8.837819	2.606583	-1.336786
H	-9.310840	1.096322	-1.578558
H	-9.901377	2.510711	0.554152
H	-10.064616	0.916938	0.452436
H	-9.405757	2.586797	2.837008
H	-9.756252	1.029201	2.880267
H	-2.818897	-1.772424	4.517012
H	-2.480405	-2.336154	5.982051
H	-4.226630	-1.241951	6.871743
H	-4.415549	-0.506192	5.460243
H	-4.949892	-4.218046	3.541663
H	-4.119940	-2.915750	3.128322
H	-5.875432	-1.604083	3.721339
H	-6.708996	-2.914836	4.142905
H	-3.747620	-4.273101	6.819844
H	-4.660321	-5.136716	5.822323
H	-6.450682	-3.901697	6.296343
H	-5.590545	-3.195106	7.450676
H	-7.711612	-1.779316	6.099855
H	-6.780715	-1.165826	7.243770
H	-7.300964	4.225848	3.285354
H	-7.630135	4.661689	4.789623
H	-8.785223	4.059317	3.860118
H	-9.614761	-0.506852	3.709171
H	-9.002529	-1.452849	4.845529
H	-8.309310	-1.381123	3.404938
H	-4.808792	0.582599	7.381992
H	-6.171429	0.570019	8.221119
H	-5.452256	1.959286	7.883712
H	-6.572796	-2.662359	-6.206512
H	-5.374491	-2.151145	-7.137380
H	-5.044273	-4.273646	-7.711589
H	-6.039497	-4.794457	-6.561090
H	-4.783200	-2.897178	-3.330945
H	-6.111561	-3.354381	-4.097555
H	-5.148088	-5.333262	-4.317715
H	-3.711334	-4.752569	-3.893068
H	-3.155251	-1.523586	-6.023083
H	-2.923851	-2.133207	-4.555932
H	-2.146958	-3.934116	-5.581961
H	-2.744414	-3.468094	-6.995573
H	-4.467919	-6.607063	-6.249079
H	-3.497632	-5.969755	-7.344233
H	0.360678	-6.337578	0.231740
H	0.708091	-7.839378	0.671335
H	0.548019	-8.404371	-1.448236
H	-0.044171	-6.966061	-1.836875
H	-3.084903	-6.612274	0.698536
H	-1.946780	-5.704927	0.038560
H	-2.487059	-6.574294	-1.903611
H	-3.449890	-7.655625	-1.202487
H	-2.606583	-8.837819	1.336786
H	-1.096322	-9.310840	1.578558
H	-2.510711	-9.901377	-0.554152
H	-0.916938	-10.064616	-0.452436
H	-2.586797	-9.405757	-2.837008
H	-1.029201	-9.756252	-2.880267
H	1.772424	-2.818897	-4.517012
H	2.336154	-2.480405	-5.982051
H	1.241951	-4.226630	-6.871743
H	0.506192	-4.415549	-5.460243
H	4.218046	-4.949892	-3.541663
H	2.915750	-4.119940	-3.128322
H	1.604083	-5.875432	-3.721339
H	2.914836	-6.708996	-4.142905
H	4.273101	-3.747620	-6.819844
H	5.136716	-4.660321	-5.822323
H	3.901697	-6.450682	-6.296343
H	3.195106	-5.590545	-7.450676
H	1.779316	-7.711612	-6.099855
H	1.165826	-6.780715	-7.243770
H	-4.225848	-7.300964	-3.285354
H	-4.661689	-7.630135	-4.789623
H	-4.059317	-8.785223	-3.860118
H	0.506852	-9.614761	-3.709171
H	1.452849	-9.002529	-4.845529
H	1.381123	-8.309310	-3.404938
H	-0.582599	-4.808792	-7.381992
H	-0.570019	-6.171429	-8.221119
H	-1.959286	-5.452256	-7.883712
H	6.572796	2.662359	-6.206512
H	5.374491	2.151145	-7.137380
H	5.044273	4.273646	-7.711589
H	6.039497	4.794457	-6.561090
H	4.783200	2.897178	-3.330945
H	6.111561	3.354381	-4.097555
H	5.148088	5.333262	-4.317715
H	3.711334	4.752569	-3.893068
H	3.155251	1.523586	-6.023083
H	2.923851	2.133207	-4.555932
H	2.146958	3.934116	-5.581961
H	2.744414	3.468094	-6.995573
H	4.467919	6.607063	-6.249079
H	3.497632	5.969755	-7.344233
H	-0.360678	6.337578	0.231740
H	-0.708091	7.839378	0.671335
H	-0.548019	8.404371	-1.448236
H	0.044171	6.966061	-1.836875
H	3.084903	6.612274	0.698536
H	1.946780	5.704927	0.038560
H	2.487059	6.574294	-1.903611
H	3.449890	7.655625	-1.202487
H	2.606583	8.837819	1.336786
H	1.096322	9.310840	1.578558
H	2.510711	9.901377	-0.554152
H	0.916938	10.064616	-0.452436
H	2.586797	9.405757	-2.837008
H	1.029201	9.756252	-2.880267
H	-1.772424	2.818897	-4.517012
H	-2.336154	2.480405	-5.982051
H	-1.241951	4.226630	-6.871743
H	-0.506192	4.415549	-5.460243
H	-4.218046	4.949892	-3.541663
H	-2.915750	4.119940	-3.128322
H	-1.604083	5.875432	-3.721339
H	-2.914836	6.708996	-4.142905
H	-4.273101	3.747620	-6.819844
H	-5.136716	4.660321	-5.822323
H	-3.901697	6.450682	-6.296343
H	-3.195106	5.590545	-7.450676
H	-1.779316	7.711612	-6.099855
H	-1.165826	6.780715	-7.243770
H	4.225848	7.300964	-3.285354
H	4.661689	7.630135	-4.789623
H	4.059317	8.785223	-3.860118
H	-0.506852	9.614761	-3.709171
H	-1.452849	9.002529	-4.845529
H	-1.381123	8.309310	-3.404938
H	0.582599	4.808792	-7.381992
H	0.570019	6.171429	-8.221119
H	1.959286	5.452256	-7.883712

CBr_4

C	1.163701	-0.167845	-0.195136
Br	-0.257817	-0.712171	1.033949
Br	2.886954	-0.173995	0.731156
Br	1.234064	-1.422615	-1.694554
Br	0.791604	1.637402	-0.851094

$(CHBr_3\cdots4I^-)@[L_4(Cu_3I_4)_4]^{8+}$ calculations

$(CHBr_3\cdots4I^-)@[L_4(Cu_3I_4)_4]^{8+}$

I	-7.863741	1.793539	4.320780
I	-4.961662	-0.508888	6.901356
I	-7.647195	-2.498108	3.666988
I	-4.063215	-0.082494	2.693897
Cu	-5.408515	0.961675	4.788410
Cu	-5.276586	-1.629963	4.484138
Cu	-6.747463	-0.122143	2.944324
N	-4.410478	2.680063	5.362526
N	-3.189814	4.820353	6.222083
N	4.244699	2.761048	5.323458
N	3.129162	4.817919	6.185122
N	0.095673	7.545942	-0.378369
N	0.001256	8.389534	2.059496
C	-5.068222	3.145629	6.608849
H	-4.904676	2.501141	7.315868
H	-6.026155	3.195184	6.466334
C	-4.530745	4.570231	7.065735
H	-4.342207	4.575033	8.016150
H	-5.187387	5.258576	6.873967
C	-2.953238	2.506981	5.677429
H	-2.854397	1.701255	6.209659
H	-2.480104	2.359279	4.842787
C	-2.329291	3.545884	6.348094
H	-1.451809	3.702907	5.968457
H	-2.220632	3.314468	7.283584
C	-4.434538	3.727952	4.370552
H	-5.355397	3.984799	4.208841
H	-4.077073	3.377882	3.538567
C	-3.612114	5.016881	4.771801
H	-2.832480	5.113923	4.200820
H	-4.161210	5.811825	4.681932
C	-2.565144	6.142497	6.744681
H	-3.198259	6.858371	6.577791
H	-2.464276	6.066205	7.705811
C	4.958572	3.176619	6.472609
H	5.896327	3.304587	6.259232
H	4.889807	2.509167	7.172722
C	4.308460	4.559836	6.960275
H	4.078098	4.503846	7.899845
H	4.943298	5.283041	6.843025
C	4.134483	3.830315	4.282202
H	5.022110	4.080868	3.982331
H	3.646727	3.484539	3.517445
C	3.451768	4.999918	4.798792
H	4.020050	5.780094	4.698232
H	2.637864	5.153036	4.293880
C	2.867145	2.397620	5.691696
H	2.876084	1.684104	6.348004
H	2.387905	2.083687	4.907197
C	2.171144	3.629843	6.266822
H	1.364213	3.820330	5.765107
H	1.924790	3.468173	7.191777
C	2.453734	6.088289	6.785796
H	2.360134	5.952471	7.741779
H	3.059238	6.835699	6.660808
C	0.044915	9.050453	-0.308573
H	-0.764340	9.360095	-0.746043
H	0.801504	9.414847	-0.794980
C	0.070827	9.575061	1.117650
H	0.886806	10.076754	1.272223
H	-0.684259	10.164980	1.268645
C	-1.026145	7.011053	0.299238
H	-0.942687	6.046127	0.342445
H	-1.831107	7.218500	-0.202480
C	-1.163719	7.587852	1.760570
H	-1.963582	8.130649	1.827255
H	-1.233772	6.859361	2.396441
C	1.218936	7.203321	0.339401
H	1.989733	7.618314	-0.076915
H	1.337610	6.242197	0.284288
C	1.184134	7.592550	1.760929
H	1.182899	6.795236	2.314029
H	1.980628	8.103921	1.974713
C	-0.030781	8.954609	3.491643
H	0.734709	9.536645	3.610069
H	-0.829379	9.496710	3.588940
C	-1.212906	6.564320	6.160264
C	-0.021390	6.108377	6.710955
C	1.093581	6.495264	6.239847
C	1.131900	7.489269	5.124943
C	-0.020197	7.914103	4.586017
C	-1.386631	7.386807	5.102963
C	-0.020165	5.253408	7.975102
H	-0.067315	5.821978	8.745722
H	0.785232	4.732784	8.007874
H	-0.780368	4.666156	7.962610
C	2.464068	8.138725	4.758778
H	3.169738	7.492282	4.849848
H	2.631427	8.878170	5.346969
H	2.430633	8.449636	3.852421
C	-2.611347	8.114775	4.677741
H	-3.343569	7.497236	4.609921
H	-2.459014	8.526029	3.824601
H	-2.823453	8.791152	5.326085
I	7.863741	-2.387559	4.001036

I	4.961662	-0.504303	6.901707
I	7.647195	1.953597	4.007426
I	4.063215	-0.285960	2.678337
Cu	5.408515	-1.636475	4.589720
Cu	5.276586	0.971294	4.683065
Cu	6.747463	-0.284852	2.931881
N	4.410478	-3.422179	4.895865
N	3.189814	-5.668284	5.419978
N	-4.244699	-3.496282	4.844937
N	-3.129162	-5.660259	5.383818
N	-0.095673	-7.358527	-1.518168
N	-0.001256	-8.563006	0.763073
C	5.068222	-4.071846	6.056903
H	4.904676	-3.542360	6.853699
H	6.026155	-4.099153	5.908509
C	4.530745	-5.549354	6.291853
H	4.342207	-5.698619	7.230486
H	5.187387	-6.200536	5.997647
C	2.953238	-3.298994	5.233425
H	2.854397	-2.583567	5.881983
H	2.480104	-3.026095	4.430947
C	2.329291	-4.427796	5.738318
H	1.451809	-4.525267	5.339218
H	2.220632	-4.341320	6.698118
C	4.434538	-4.307046	3.756086
H	5.355397	-4.536316	3.557200
H	4.077073	-3.834536	2.987007
C	3.612114	-5.641999	3.956677
H	2.832480	-5.651091	3.377580
H	4.161210	-6.414034	3.746976
C	2.565144	-7.054518	5.735457
H	3.198259	-7.736691	5.461653
H	2.464276	-7.125262	6.697011
C	-4.958572	-4.081758	5.917534
H	-5.896327	-4.175792	5.687180
H	-4.889807	-3.528526	6.710998
C	-4.308460	-5.523044	6.189200
H	-4.078098	-5.610574	7.126359
H	-4.943298	-6.220011	5.963344
C	-4.134483	-4.394784	3.653198
H	-5.022110	-4.596826	3.318717
H	-3.646727	-3.936742	2.949913
C	-3.451768	-5.629338	3.985934
H	-4.020050	-6.385151	3.767912
H	-2.637864	-5.703900	3.463611
C	-2.867145	-3.193074	5.264155
H	-2.876084	-2.587652	6.021327
H	-2.387905	-2.763502	4.536515
C	-2.171144	-4.498421	5.645224
H	-1.364213	-4.610403	5.120379
H	-1.924790	-4.479277	6.584006
C	-2.453734	-7.007193	5.784337
H	-2.360134	-7.018319	6.749855
H	-3.059238	-7.726907	5.547152
C	-0.044915	-8.856156	-1.677957
H	0.764340	-9.095676	-2.157423
H	-0.801504	-9.142350	-2.214116
C	-0.070827	-9.591532	-0.348089
H	-0.886806	-10.110895	-0.271600
H	0.684259	-10.197551	-0.288552
C	1.026145	-6.932894	-0.767106
H	0.942687	-5.985758	-0.577677
H	1.831107	-7.061638	-1.294535
C	1.163719	-7.725192	0.589526
H	1.963582	-8.271817	0.572899
H	1.233772	-7.101861	1.328776
C	-1.218936	-7.129033	-0.756647
H	-1.989733	-7.475896	-1.231224
H	-1.337610	-6.170704	-0.664972
C	-1.184134	-7.729890	0.589167
H	-1.182899	-7.025951	1.257072
H	-1.980628	-8.267822	0.722706
C	0.030781	-9.339279	2.092642
H	-0.734709	-9.932556	2.121188
H	0.829379	-9.889871	2.106377
C	1.212906	-7.382571	5.093694
C	0.021390	-7.015667	5.707312
C	-1.093581	-7.326419	5.182852
C	-1.131900	-8.139336	3.929767
C	0.020197	-8.477281	3.332508
C	1.386631	-8.034723	3.923623
C	0.020165	-6.362863	7.086763
H	0.067315	-7.042001	7.761966
H	-0.785232	-5.853277	7.198320
H	0.780368	-5.780541	7.163713
C	-2.464068	-8.725561	3.469104
H	-3.169738	-8.100483	3.657412
H	-2.631427	-9.545846	3.938017
H	-2.430633	-8.895038	2.526011
C	2.611347	-8.689567	3.392651

H	3.343569	-8.068897	3.419522
H	2.459014	-8.966313	2.486898
H	2.823453	-9.456665	3.930607
I	-2.096653	-7.503960	-4.727798
I	0.002299	-4.400745	-7.247135
I	2.232352	-7.312799	-4.387585
I	-0.102030	-3.827282	-2.963213
Cu	-1.302868	-5.015492	-5.068741
Cu	1.304426	-4.892012	-4.953218
Cu	-0.081592	-6.484444	-3.419875
N	-3.060030	-3.986702	-5.432771
N	-5.259631	-2.716685	-6.031529
N	-3.137800	4.639835	-4.727807
N	-5.254386	3.581099	-5.513140
N	-7.473994	0.041564	0.988292
N	-8.501019	0.127865	-1.378460
C	-3.619275	-4.550481	-6.686605
H	-3.030573	-4.329910	-7.426042
H	-3.657818	-5.516749	-6.614194
C	-5.074566	-3.988115	-6.991539
H	-5.151825	-3.727895	-7.921687
H	-5.746286	-4.661428	-6.798623
C	-2.911464	-2.508757	-5.647886
H	-2.148667	-2.365053	-6.230743
H	-2.700549	-2.099599	-4.793249
C	-3.998481	-1.841676	-6.188088
H	-4.126100	-0.996526	-5.731811
H	-3.839071	-1.660864	-7.127448
C	-4.029230	-4.092203	-4.368728
H	-4.272997	-5.024170	-4.258646
H	-3.616739	-3.796999	-3.540940
C	-5.345001	-3.249161	-4.606938
H	-5.398223	-2.515773	-3.972451
H	-6.130778	-3.808113	-4.499022
C	-6.617774	-2.061788	-6.402935
H	-7.318838	-2.709910	-6.230864
H	-6.614992	-1.887696	-7.356586
C	-3.639785	5.436583	-5.784247
H	-3.751110	6.354640	-5.490877
H	-3.027661	5.425127	-6.536279
C	-5.056160	4.817398	-6.213485
H	-5.071976	4.659467	-7.169414
H	-5.768320	5.437269	-5.993525
C	-4.124558	4.444558	-3.619713
H	-4.351516	5.305345	-3.234855
H	-3.721475	3.902088	-2.922884
C	-5.330146	3.796306	-4.096434
H	-6.100383	4.350752	-3.893811
H	-5.444318	2.945493	-3.644877
C	-2.803509	3.296402	-5.226574
H	-2.142114	3.359362	-5.932633
H	-2.430671	2.760738	-4.507047
C	-4.075999	2.638989	-5.757743
H	-4.227675	1.795155	-5.305992
H	-3.985328	2.464615	-6.708396
C	-6.566860	2.945919	-6.065238
H	-6.504331	2.926064	-7.033126
H	-7.302563	3.535812	-5.837982
C	-8.979447	-0.012488	1.029418
H	-9.254829	-0.854447	1.426180
H	-9.305690	0.702799	1.598392
C	-9.611279	0.118734	-0.346651
H	-10.123297	0.941172	-0.399963
H	-10.210993	-0.626104	-0.509493
C	-6.992331	-1.022361	0.188418
H	-6.033508	-0.930250	0.078463
H	-7.160917	-1.864330	0.641612
C	-7.678878	-1.051784	-1.231054
H	-8.225179	-1.847404	-1.317074
H	-7.000993	-1.069052	-1.923956
C	-7.187101	1.218120	0.334296
H	-7.569141	1.952609	0.838517
H	-6.224573	1.337847	0.325065
C	-7.683590	1.289234	-1.052028
H	-6.930771	1.334690	-1.662625
H	-8.209773	2.096689	-1.164957
C	-9.173652	0.201521	-2.761761
H	-9.763024	0.970402	-2.776878
H	-9.721593	-0.590507	-2.878171
C	-6.993807	-0.760372	-5.686736
C	-6.581182	0.472194	-6.178036
C	-6.931020	1.545852	-5.595235
C	-7.837119	1.493515	-4.408318
C	-8.219622	0.301328	-3.928074
C	-7.733279	-1.018759	-4.586296
C	-5.825094	0.574499	-7.499742
H	-6.450770	0.582770	-8.226248
H	-5.308485	1.383069	-7.510494
H	-5.238600	-0.181027	-7.589937
C	-8.456764	2.790189	-3.893325

H	-7.819147	3.504488	-3.979206
H	-9.238906	2.997482	-4.409115
H	-8.697659	2.686134	-2.971149
C	-8.426704	-2.276471	-4.201585
H	-7.805792	-3.008127	-4.236944
H	-8.771708	-2.191836	-3.310524
H	-9.150549	-2.442600	-4.810909
I	2.096653	8.177734	-3.528561
I	-0.002299	5.493690	-6.490471
I	-2.232352	7.937064	-3.221372
I	0.102030	4.275490	-2.343564
Cu	1.302868	5.770046	-4.243931
Cu	-1.304426	5.630436	-4.148528
Cu	0.081592	6.971193	-2.390873
N	3.060030	4.808573	-4.760164
N	5.259631	3.644370	-5.545075
N	3.137800	-3.824846	-5.375132
N	5.254386	-2.659005	-5.990344
N	7.473994	-0.149225	0.973701
N	8.501019	0.125361	-1.378652
C	3.619275	5.556452	-5.913691
H	3.030573	5.450883	-6.678069
H	3.657818	6.500473	-5.695193
C	5.074566	5.046993	-6.300592
H	5.151825	4.931235	-7.259492
H	5.746286	5.683142	-6.007535
C	2.911464	3.380524	-5.197511
H	2.148667	3.327120	-5.795442
H	2.700549	2.846169	-4.415028
C	3.998481	2.803342	-5.832867
H	4.126100	1.898639	-5.510407
H	3.839071	2.767470	-6.788797
C	4.029230	4.751051	-3.692452
H	4.272997	5.655441	-3.441937
H	3.616739	4.333408	-2.919178
C	5.345001	3.954034	-4.056083
H	5.398223	3.132695	-3.540492
H	6.130778	4.490077	-3.864430
C	6.617774	3.053564	-6.011746
H	7.318838	3.667985	-5.743123
H	6.614992	3.026507	-6.980779
C	3.639785	-4.451689	-6.540439
H	3.751110	-5.403680	-6.390079
H	3.027661	-4.326014	-7.281984
C	5.056160	-3.774435	-6.870534
H	5.071976	-3.472984	-7.791333
H	5.768320	-4.420545	-6.747388
C	4.124558	-3.800334	-4.250230
H	4.351516	-4.709633	-4.000737
H	3.721475	-3.370131	-3.479017
C	5.330146	-3.087132	-4.622835
H	6.100383	-3.665940	-4.506877
H	5.444318	-2.314875	-4.047157
C	2.803509	-2.421194	-5.663820
H	2.142114	-2.376060	-6.371242
H	2.430671	-2.001168	-4.871208
C	4.075999	-1.690657	-6.088847
H	4.227675	-0.925328	-5.514037
H	3.985328	-1.373757	-7.001930
C	6.566860	-1.947261	-6.439438
H	6.504331	-1.780461	-7.393051
H	7.302563	-2.564851	-6.304522
C	8.979447	-0.102055	1.022568
H	9.254829	0.669782	1.542743
H	9.305690	-0.895542	1.476161
C	9.611279	-0.022508	-0.357453
H	10.123297	-0.827276	-0.535203
H	10.210993	0.738430	-0.405142
C	6.992331	1.023955	0.344907
H	6.033508	0.949635	0.222225
H	7.160917	1.787222	0.920860
C	7.678878	1.268878	-1.053585
H	8.225179	2.068327	-1.017623
H	7.000993	1.391307	-1.735804
C	7.187101	-1.212654	0.148406
H	7.569141	-2.015272	0.535078
H	6.224573	-1.329585	0.121077
C	7.683590	-1.072140	-1.232611
H	6.930771	-1.024221	-1.843019
H	8.209773	-1.853033	-1.467006
C	9.173652	0.262904	-2.757067
H	9.763024	-0.494738	-2.888923
H	9.721593	1.063423	-2.751689
C	6.993807	1.658377	-5.501765
C	6.581182	0.514850	-6.174774
C	6.931020	-0.634942	-5.762008
C	7.837119	-0.763695	-4.580935
C	8.219622	0.341604	-3.924994
C	7.733279	1.746429	-4.374832
C	5.825094	0.614711	-7.496667

H	6.450770	0.717008	-8.215983
H	5.308485	-0.182822	-7.630243
H	5.238600	1.375166	-7.470929
C	8.456764	-2.123599	-4.269100
H	7.819147	-2.816532	-4.462597
H	9.238906	-2.250051	-4.810413
H	8.697659	-2.160978	-3.341826
C	8.426704	2.931018	-3.803350
H	7.805792	3.659542	-3.727043
H	8.771708	2.711874	-2.935519
H	9.150549	3.187867	-4.380326
I	-0.031039	4.104697	3.142251
C	0.000000	0.019939	0.016364
Br	0.000000	-1.663073	1.013232
Br	-1.602099	0.105515	-1.102665
Br	1.602099	0.105515	-1.102665
H	-0.000000	0.855494	0.714768
I	0.031039	-4.492636	2.484781
I	-4.311218	0.202899	-2.783154
I	4.311218	0.264795	-2.778421

$4I^{-}@[L_4(Cu_3I_4)_4]^{8+}$

I	2.090530	7.865371	4.156645
I	-0.006162	4.961659	6.905271
I	-2.238304	7.645455	3.831999
I	0.098867	4.063294	2.677548
Cu	1.298657	5.409528	4.686344
Cu	-1.308534	5.275569	4.580573
Cu	0.076339	6.747524	2.930269
N	3.056595	4.412859	5.127760
N	5.257146	3.193908	5.821615
N	3.141103	-4.242255	5.082631
N	5.256821	-3.125071	5.784948
N	7.474066	-0.089854	-0.967449
N	8.501018	0.005362	1.398993
C	3.615328	5.071038	6.334955
H	3.026754	4.907033	7.089058
H	3.653125	6.029001	6.189076
C	5.071038	4.534694	6.681883
H	5.148443	4.346216	7.629165
H	5.742246	5.191859	6.438187
C	2.909164	2.955504	5.454944
H	2.146444	2.856068	6.047062
H	2.698617	2.482206	4.633994
C	3.996666	2.332403	6.044439
H	4.124968	1.455021	5.653934
H	3.837341	2.223620	6.994851
C	4.025776	4.437673	4.058771
H	4.268827	5.358722	3.877946
H	3.613564	4.079887	3.255902
C	5.342188	3.616273	4.360570
H	5.396016	2.836682	3.783852
H	6.127537	4.165981	4.210348
C	6.615775	2.570295	6.241877
H	7.316346	3.203955	6.020887
H	6.613072	2.469425	7.206026
C	3.643644	-4.955737	6.196748
H	3.755699	-5.893405	5.974235
H	3.031467	-4.887448	6.945717
C	5.059513	-4.304522	6.577523
H	5.075149	-4.074149	7.518627
H	5.772166	-4.938806	6.405469
C	4.127775	-4.131271	3.962873
H	4.355424	-5.018721	3.644771
H	3.724313	-3.643828	3.226709
C	5.332831	-3.447618	4.388776
H	6.103511	-4.015300	4.229020
H	5.446370	-2.633625	3.873659
C	2.805741	-2.864962	5.477508
H	2.144353	-2.874416	6.186312
H	2.432529	-2.386012	4.719231
C	4.077688	-2.167971	5.957002
H	4.228736	-1.360922	5.442223
H	3.986825	-1.921687	6.891591
C	6.568768	-2.448621	6.287006
H	6.506167	-2.355069	7.250562
H	7.304943	-3.053552	6.105391
C	8.979479	-0.037924	-1.012577
H	9.254232	0.771544	-1.472384
H	9.306312	-0.794259	-1.525353
C	9.611331	-0.063345	0.369492
H	10.123984	-0.878925	0.485360
H	10.210457	0.692208	0.475065
C	6.991530	1.031588	-0.251029
H	6.032772	0.947383	-0.134371
H	7.159489	1.836681	-0.767105
C	7.677970	1.169696	1.162067
H	8.223648	1.969985	1.187169
H	7.000031	1.239222	1.851635
C	7.188048	-1.213340	-0.225644
H	7.570688	-1.983840	-0.672391
H	6.225612	-1.332764	-0.207310
C	7.684509	-1.178152	1.162067
H	6.931690	-1.177503	1.774352
H	8.211312	-1.974236	1.336236
C	9.173625	0.037923	2.783883
H	9.763593	-0.727109	2.857584
H	9.720945	0.836947	2.839561
C	6.992861	1.218350	5.626997
C	6.581163	0.026514	6.210851
C	6.931869	-1.088185	5.711614
C	7.837998	-1.125799	4.524162
C	8.219604	0.026596	3.954411
C	7.732197	1.392651	4.510059
C	5.825077	0.024700	7.536510
H	6.450715	0.072337	8.261532
H	5.309095	-0.781099	7.608885
H	5.237991	0.784446	7.568833
C	8.458679	-2.457484	4.109541
H	7.821612	-3.163650	4.249638
H	9.240951	-2.624234	4.639636
H	8.699549	-2.423861	3.182116
C	8.424669	2.617906	4.030566
H	7.803187	3.349644	4.010032
H	8.769791	2.465842	3.148552
H	9.148349	2.830576	4.625448
I	-2.090530	-7.865371	4.156645
I	0.006162	-4.961659	6.905271
I	2.238304	-7.645455	3.831999
I	-0.098867	-4.063294	2.677548
Cu	-1.298657	-5.409528	4.686344
Cu	1.308534	-5.275569	4.580573
Cu	-0.076339	-6.747524	2.930269
N	-3.056595	-4.412859	5.127760
N	-5.257146	-3.193908	5.821615
N	-3.141103	4.242255	5.082631
N	-5.256821	3.125071	5.784948
N	-7.474066	0.089854	-0.967449
N	-8.501018	-0.005362	1.398993
C	-3.615328	-5.071038	6.334955
H	-3.026754	-4.907033	7.089058
H	-3.653125	-6.029001	6.189076
C	-5.071038	-4.534694	6.681883
H	-5.148443	-4.346216	7.629165
H	-5.742246	-5.191859	6.438187
C	-2.909164	-2.955504	5.454944
H	-2.146444	-2.856068	6.047062
H	-2.698617	-2.482206	4.633994
C	-3.996666	-2.332403	6.044439
H	-4.124968	-1.455021	5.653934
H	-3.837341	-2.223620	6.994851
C	-4.025776	-4.437673	4.058771
H	-4.268827	-5.358722	3.877946
H	-3.613564	-4.079887	3.255902
C	-5.342188	-3.616273	4.360570
H	-5.396016	-2.836682	3.783852
H	-6.127537	-4.165981	4.210348
C	-6.615775	-2.570295	6.241877
H	-7.316346	-3.203955	6.020887
H	-6.613072	-2.469425	7.206026
C	-3.643644	4.955737	6.196748
H	-3.755699	5.893405	5.974235
H	-3.031467	4.887448	6.945717
C	-5.059513	4.304522	6.577523
H	-5.075149	4.074149	7.518627
H	-5.772166	4.938806	6.405469
C	-4.127775	4.131271	3.962873
H	-4.355424	5.018721	3.644771
H	-3.724313	3.643828	3.226709
C	-5.332831	3.447618	4.388776
H	-6.103511	4.015300	4.229020
H	-5.446370	2.633625	3.873659
C	-2.805741	2.864962	5.477508
H	-2.144353	2.874416	6.186312
H	-2.432529	2.386012	4.719231
C	-4.077688	2.167971	5.957002
H	-4.228736	1.360922	5.442223
H	-3.986825	1.921687	6.891591
C	-6.568768	2.448621	6.287006
H	-6.506167	2.355069	7.250562
H	-7.304943	3.053552	6.105391
C	-8.979479	0.037924	-1.012577
H	-9.254232	-0.771544	-1.472384
H	-9.306312	0.794259	-1.525353
C	-9.611331	0.063345	0.369492
H	-10.123984	0.878925	0.485360
H	-10.210457	-0.692208	0.475065
C	-6.991530	-1.031588	-0.251029
H	-6.032772	-0.947383	-0.134371
H	-7.159489	-1.836681	-0.767105
C	-7.677970	-1.169696	1.162067
H	-8.223648	-1.969985	1.187169
H	-7.000031	-1.239222	1.851635
C	-7.188048	1.213340	-0.225644
H	-7.570688	1.983840	-0.672391
H	-6.225612	1.332764	-0.207310
C	-7.684509	1.178152	1.162067
H	-6.931690	1.177503	1.774352
H	-8.211312	1.974236	1.336236
C	-9.173625	-0.037923	2.783883
H	-9.763593	0.727109	2.857584
H	-9.720945	-0.836947	2.839561
C	-6.992861	-1.218350	5.626997
C	-6.581163	-0.026514	6.210851
C	-6.931869	1.088185	5.711614
C	-7.837998	1.125799	4.524162
C	-8.219604	-0.026596	3.954411
C	-7.732197	-1.392651	4.510059
C	-5.825077	-0.024700	7.536510
H	-6.450715	-0.072337	8.261532
H	-5.309095	0.781099	7.608885
H	-5.237991	-0.784446	7.568833
C	-8.458679	2.457484	4.109541
H	-7.821612	3.163650	4.249638
H	-9.240951	2.624234	4.639636
H	-8.699549	2.423861	3.182116
C	-8.424669	-2.617906	4.030566
H	-7.803187	-3.349644	4.010032
H	-8.769791	-2.465842	3.148552
H	-9.148349	-2.830576	4.625448
I	-7.865371	2.090530	-4.156645
I	-4.961659	-0.006162	-6.905271
I	-7.645455	-2.238304	-3.831999
I	-4.063294	0.098867	-2.677548
Cu	-5.409528	1.298657	-4.686344
Cu	-5.275569	-1.308534	-4.580573
Cu	-6.747524	0.076339	-2.930269
N	-4.412859	3.056595	-5.127760
N	-3.193908	5.257146	-5.821615
N	4.242255	3.141103	-5.082631
N	3.125071	5.256821	-5.784948
N	0.089854	7.474066	0.967449
N	-0.005362	8.501018	-1.398993
C	-5.071038	3.615328	-6.334955
H	-4.907033	3.026754	-7.089058
H	-6.029001	3.653125	-6.189076
C	-4.534694	5.071038	-6.681883
H	-4.346216	5.148443	-7.629165
H	-5.191859	5.742246	-6.438187
C	-2.955504	2.909164	-5.454944
H	-2.856068	2.146444	-6.047062
H	-2.482206	2.698617	-4.633994
C	-2.332403	3.996666	-6.044439
H	-1.455021	4.124968	-5.653934
H	-2.223620	3.837341	-6.994851
C	-4.437673	4.025776	-4.058771
H	-5.358722	4.268827	-3.877946
H	-4.079887	3.613564	-3.255902
C	-3.616273	5.342188	-4.360570
H	-2.836682	5.396016	-3.783852
H	-4.165981	6.127537	-4.210348
C	-2.570295	6.615775	-6.241877
H	-3.203955	7.316346	-6.020887
H	-2.469425	6.613072	-7.206026
C	4.955737	3.643644	-6.196748
H	5.893405	3.755699	-5.974235
H	4.887448	3.031467	-6.945717
C	4.304522	5.059513	-6.577523
H	4.074149	5.075149	-7.518627
H	4.938806	5.772166	-6.405469
C	4.131271	4.127775	-3.962873
H	5.018721	4.355424	-3.644771
H	3.643828	3.724313	-3.226709
C	3.447618	5.332831	-4.388776
H	4.015300	6.103511	-4.229020
H	2.633625	5.446370	-3.873659
C	2.864962	2.805741	-5.477508
H	2.874416	2.144353	-6.186312
H	2.386012	2.432529	-4.719231
C	2.167971	4.077688	-5.957002
H	1.360922	4.228736	-5.442223
H	1.921687	3.986825	-6.891591
C	2.448621	6.568768	-6.287006
H	2.355069	6.506167	-7.250562
H	3.053552	7.304943	-6.105391
C	0.037924	8.979479	1.012577
H	-0.771544	9.254232	1.472384
H	0.794259	9.306312	1.525353

C	0.063345	9.611331	-0.369492
H	0.878925	10.123984	-0.485360
H	-0.692208	10.210457	-0.475065
C	-1.031588	6.991530	0.251029
H	-0.947383	6.032772	0.134371
H	-1.836681	7.159489	0.767105
C	-1.169696	7.677970	-1.162067
H	-1.969985	8.223648	-1.187169
H	-1.239222	7.000031	-1.851635
C	1.213340	7.188048	0.225644
H	1.983840	7.570688	0.672391
H	1.332764	6.225612	0.207310
C	1.178152	7.684509	-1.162067
H	1.177503	6.931690	-1.774352
H	1.974236	8.211312	-1.336236
C	-0.037923	9.173625	-2.783883
H	0.727109	9.763593	-2.857584
H	-0.836947	9.720945	-2.839561
C	-1.218350	6.992861	-5.626997
C	-0.026514	6.581163	-6.210851
C	1.088185	6.931869	-5.711614
C	1.125799	7.837998	-4.524162
C	-0.026596	8.219604	-3.954411
C	-1.392651	7.732197	-4.510059
C	-0.024700	5.825077	-7.536510
H	-0.072337	6.450715	-8.261532
H	0.781099	5.309095	-7.608885
H	-0.784446	5.237991	-7.568833
C	2.457484	8.458679	-4.109541
H	3.163650	7.821612	-4.249638
H	2.624234	9.240951	-4.639636
H	2.423861	8.699549	-3.182116
C	-2.617906	8.424669	-4.030566
H	-3.349644	7.803187	-4.010032
H	-2.465842	8.769791	-3.148552
H	-2.830576	9.148349	-4.625448
I	7.865371	-2.090530	-4.156645
I	4.961659	0.006162	-6.905271
I	7.645455	2.238304	-3.831999
I	4.063294	-0.098867	-2.677548
Cu	5.409528	-1.298657	-4.686344
Cu	5.275569	1.308534	-4.580573
Cu	6.747524	-0.076339	-2.930269
N	4.412859	-3.056595	-5.127760
N	3.193908	-5.257146	-5.821615
N	-4.242255	-3.141103	-5.082631
N	-3.125071	-5.256821	-5.784948
N	-0.089854	-7.474066	0.967449
N	0.005362	-8.501018	-1.398993
C	5.071038	-3.615328	-6.334955
H	4.907033	-3.026754	-7.089058
H	6.029001	-3.653125	-6.189076
C	4.534694	-5.071038	-6.681883
H	4.346216	-5.148443	-7.629165
H	5.191859	-5.742246	-6.438187
C	2.955504	-2.909164	-5.454944
H	2.856068	-2.146444	-6.047062
H	2.482206	-2.698617	-4.633994
C	2.332403	-3.996666	-6.044439
H	1.455021	-4.124968	-5.653934
H	2.223620	-3.837341	-6.994851
C	4.437673	-4.025776	-4.058771
H	5.358722	-4.268827	-3.877946
H	4.079887	-3.613564	-3.255902
C	3.616273	-5.342188	-4.360570
H	2.836682	-5.396016	-3.783852
H	4.165981	-6.127537	-4.210348
C	2.570295	-6.615775	-6.241877
H	3.203955	-7.316346	-6.020887
H	2.469425	-6.613072	-7.206026
C	-4.955737	-3.643644	-6.196748
H	-5.893405	-3.755699	-5.974235
H	-4.887448	-3.031467	-6.945717
C	-4.304522	-5.059513	-6.577523
H	-4.074149	-5.075149	-7.518627
H	-4.938806	-5.772166	-6.405469
C	-4.131271	-4.127775	-3.962873
H	-5.018721	-4.355424	-3.644771
H	-3.643828	-3.724313	-3.226709
C	-3.447618	-5.332831	-4.388776
H	-4.015300	-6.103511	-4.229020
H	-2.633625	-5.446370	-3.873659
C	-2.864962	-2.805741	-5.477508
H	-2.874416	-2.144353	-6.186312
H	-2.386012	-2.432529	-4.719231
C	-2.167971	-4.077688	-5.957002
H	-1.360922	-4.228736	-5.442223
H	-1.921687	-3.986825	-6.891591
C	-2.448621	-6.568768	-6.287006
H	-2.355069	-6.506167	-7.250562
H	-3.053552	-7.304943	-6.105391
C	-0.037924	-8.979479	1.012577
H	0.771544	-9.254232	1.472384
H	-0.794259	-9.306312	1.525353
C	-0.063345	-9.611331	-0.369492
H	-0.878925	-10.123984	-0.485360
H	0.692208	-10.210457	-0.475065
C	1.031588	-6.991530	0.251029
H	0.947383	-6.032772	0.134371
H	1.836681	-7.159489	0.767105
C	1.169696	-7.677970	-1.162067
H	1.969985	-8.223648	-1.187169
H	1.239222	-7.000031	-1.851635
C	-1.213340	-7.188048	0.225644
H	-1.983840	-7.570688	0.672391
H	-1.332764	-6.225612	0.207310
C	-1.178152	-7.684509	-1.162067
H	-1.177503	-6.931690	-1.774352
H	-1.974236	-8.211312	-1.336236
C	0.037923	-9.173625	-2.783883
H	-0.727109	-9.763593	-2.857584
H	0.836947	-9.720945	-2.839561
C	1.218350	-6.992861	-5.626997
C	0.026514	-6.581163	-6.210851
C	-1.088185	-6.931869	-5.711614
C	-1.125799	-7.837998	-4.524162
C	0.026596	-8.219604	-3.954411
C	1.392651	-7.732197	-4.510059
C	0.024700	-5.825077	-7.536510
H	0.072337	-6.450715	-8.261532
H	-0.781099	-5.309095	-7.608885
H	0.784446	-5.237991	-7.568833
C	-2.457484	-8.458679	-4.109541
H	-3.163650	-7.821612	-4.249638
H	-2.624234	-9.240951	-4.639636
H	-2.423861	-8.699549	-3.182116
C	2.617906	-8.424669	-4.030566
H	3.349644	-7.803187	-4.010032
H	2.465842	-8.769791	-3.148552
H	2.830576	-9.148349	-4.625448
I	4.311193	0.034395	2.805319
I	-4.311193	-0.034395	2.805319
I	-0.034395	4.311193	-2.805319
I	0.034395	-4.311193	-2.805319

$CHBr_3$

C	0.000000	0.019939	0.016364
Br	0.000000	-1.663073	1.013232
Br	-1.602099	0.105515	-1.102665
Br	1.602099	0.105515	-1.102665
H	-0.000000	0.855494	0.714768

$4I^{-}\cdots$halomethane complexes

$4I^{-}\cdots CBr_4$ calculations

$4I^{-}\cdots CBr_4$

C	-0.006523	0.010878	-0.024575
Br	-1.896542	0.316633	0.392189
Br	0.688813	1.517978	-1.065458
Br	0.174211	-1.642906	-1.059437
Br	1.007066	-0.152482	1.644671
I	2.750185	-0.451511	4.626701
I	-5.233049	0.846297	1.167367
I	0.488108	-4.611517	-2.821314
I	1.966214	4.175825	-2.873107

CBr_4

C	-0.006523	0.010878	-0.024575
Br	-1.896542	0.316633	0.392189
Br	0.688813	1.517978	-1.065458
Br	0.174211	-1.642906	-1.059437
Br	1.007066	-0.152482	1.644671

$4I^{-}$

I	2.750185	-0.451511	4.626701
I	-5.233049	0.846297	1.167367
I	0.488108	-4.611517	-2.821314
I	1.966214	4.175825	-2.873107

$4I^{-}\cdots CHBr_3$ calculations

$4I^{-}\cdots CHBr_3$

C	-0.008729	0.069507	0.003862
Br	0.700453	1.534645	-1.054416
Br	0.998185	-0.185566	1.644031
Br	-1.906124	0.288755	0.351668
I	2.016275	4.190805	-3.111399
I	-5.458286	0.595652	0.927502
I	2.918837	-0.757654	4.657398
I	0.199869	-3.115038	-2.061948
H	0.103275	-0.850106	-0.594897

$CHBr_3$

C	-0.008729	0.069507	0.003862
Br	0.700453	1.534645	-1.054416
Br	0.998185	-0.185566	1.644031
Br	-1.906124	0.288755	0.351668
H	0.103275	-0.850106	-0.594897

$4I^{-}$

I	2.016275	4.190805	-3.111399
I	-5.458286	0.595652	0.927502
I	2.918837	-0.757654	4.657398
I	0.199869	-3.115038	-2.061948

$4I^{-}\cdots CCl_4$ calculations

$4I^{-}\cdots CCl_4$

C	-0.001340	-0.023910	-0.014377
Cl	-1.718819	0.258513	0.358249
Cl	0.631675	1.342578	-0.963373
Cl	0.165946	-1.525531	-0.955032
Cl	0.914686	-0.167111	1.505155
I	2.634424	-0.381104	4.663391
I	-5.166757	0.889923	1.220740
I	0.581265	-4.498459	-2.932029
I	1.897403	4.114296	-2.895690

CCl_4

C	-0.001340	-0.023910	-0.014377
Cl	-1.718819	0.258513	0.358249
Cl	0.631675	1.342578	-0.963373
Cl	0.165946	-1.525531	-0.955032
Cl	0.914686	-0.167111	1.505155

$4I^{-}$

I	2.634424	-0.381104	4.663391
I	-5.166757	0.889923	1.220740
I	0.581265	-4.498459	-2.932029
I	1.897403	4.114296	-2.895690

References

[1] P. J. Garratt, J. Ashley, J. E. Ladbury, R. O'Brien, M. B. Hursthouse, K. M. A. Malik, *Tetrahedron* **1998**, *54*, 949–968.

[2] A. Peuronen, S. Forsblom, M. Lahtinen, *Chem. Commun.* **2014**, *50*, 5469–5472.

[3] *CrysAlisPro program, Rigaku Oxford Diffraction, Oxford,* **2015**.

[4] O. V. Dolomanov, L. J. Bourhis, R. J. Gildea, J. A. K. Howard, H. Puschmann, *J. Appl. Crystallogr.* **2009**, *42*, 339–341.

[5] G. M. Sheldrick, *Acta Crystallogr. Sect. A Found. Crystallogr.* **2008**, *64*, 112–122.

[6] G. M. Sheldrick, *Acta Crystallogr. Sect. C Struct. Chem.* **2015**, *71*, 3–8.

[7] A. L. Spek, *Acta Crystallogr. Sect. C Struct. Chem.* **2015**, *71*, 9–18.

[8] D. S. Gill, J. S. Cheema, *Zeitschrift fur Phys. Chemie* **1983**, *134*, 205–214.

[9] M. J. Frisch, G. W. Trucks, H. B. Schlegel, G. E. Scuseria, M. A. Robb, J. R. Cheeseman, G. Scalmani, V. Barone, G. A. Petersson, H. Nakatsuji, X. Li, M. Caricato, A. V. Marenich, J. Bloino, B. G. Janesko, R. Gomperts, B. Mennucci, H. P. Hratchian, J. V. Ortiz, A. F. Izmaylov, J. L. Sonnenberg, D. Williams-Young, F. Ding, F. Lipparini, F. Egidi, J. Goings, B. Peng, A. Petrone, T. Henderson, D. Ranasinghe, V. G. Zakrzewski, J. Gao, N. Rega, G. Zheng, W. Liang, M. Hada, M. Ehara, K. Toyota, R. Fukuda, J. Hasegawa, M. Ishida, T. Nakajima, Y. Honda, O. Kitao, H. Nakai, T. Vreven, K. Throssell, J. Montgomery, J. A., J. E. Peralta, F. Ogliaro, M. J. Bearpark, J. J. Heyd, E. N. Brothers, K. N. Kudin, V. N. Staroverov, T. A. Keith, R. Kobayashi, J. Normand, K. Raghavachari, A. P. Rendell, J. C. Burant, S. S. Iyengar, J. Tomasi, M. Cossi, J. M. Millam, M. Klene, C. Adamo, R. Cammi, J. W. Ochterski, R. L. Martin, K. Morokuma, O. Farkas, J. B. Foresman, D. J. Fox, **2016**.
